# Molecular Epidemiology of Human Rhinoviruses and Enteroviruses Highlights Their Diversity in Sub-Saharan Africa

**DOI:** 10.3390/v7122948

**Published:** 2015-12-08

**Authors:** Arnaud G. L’Huillier, Laurent Kaiser, Tom J. Petty, Mary Kilowoko, Esther Kyungu, Philipina Hongoa, Gaël Vieille, Lara Turin, Blaise Genton, Valérie D’Acremont, Caroline Tapparel

**Affiliations:** 1 Geneva University Hospitals and Medical School, 4 rue Gabrielle-Perret-Gentil, 1211 Geneva 14, Switzerland; laurent.kaiser@hcuge.ch (L.K.); gael.vieille@hcuge.ch (G.V.); lara.turin@hcuge.ch (L.T.); caroline.tapparel@hcuge.ch (C.T.); 2 Swiss Institute of Bioinformatics, Centre Medical Universitraire, 1 rue Michel-Servet, 1211 Geneva 4, Switzerland; tompetty2@gmail.com; 3 Amana Regional Referral Hospital, PO box 25411, Dar es Salaam TZ-02, United Republic of Tanzania; mckilowoko@yahoo.com; 4 St-Francis Hospital, PO box 73, Ifakara TZ-16, United Republic of Tanzania; noemie.boillat@chuv.ch (E.K.); noemie.boillat@chuv.ch (P.H.); 5 Swiss Tropical and Public Health Institute, Socinstrasse 57, Basel 4051, Switzerland; Blaise.Genton@chuv.ch (B.G.); Valerie.Dacremont@chuv.ch (V.D.); 6 Centre Hospitalier Universitaire Vaudois, 21 rue du Bugnon, Lausanne 1011, Switzerland

**Keywords:** picornavirus, rhinovirus, enterovirus, molecular epidemiology, recombinant, new type, children, fever, Tanzania, emergent

## Abstract

Human rhinoviruses (HRVs) and enteroviruses (HEVs) belong to the *Enterovirus* genus and are the most frequent cause of infection worldwide, but data on their molecular epidemiology in Africa are scarce. To understand HRV and HEV molecular epidemiology in this setting, we enrolled febrile pediatric patients participating in a large prospective cohort assessing the causes of fever in Tanzanian children. Naso/oropharyngeal swabs were systematically collected and tested by real-time RT-PCR for HRV and HEV. Viruses from positive samples were sequenced and phylogenetic analyses were then applied to highlight the HRV and HEV types as well as recombinant or divergent strains. Thirty-eight percent (378/1005) of the enrolled children harboured an HRV or HEV infection. Although some types were predominant, many distinct types were co-circulating, including a vaccinal poliovirus, HEV-A71 and HEV-D68. Three HRV-A recombinants were identified: HRV-A36/HRV-A67, HRV-A12/HRV-A67 and HRV-A96/HRV-A61. Four divergent HRV strains were also identified: one HRV-B strain and three HRV-C strains. This is the first prospective study focused on HRV and HEV molecular epidemiology in sub-Saharan Africa. This systematic and thorough large screening with careful clinical data management confirms the wide genomic diversity of these viruses, brings new insights about their evolution and provides data about associated symptoms.

## 1. Introduction

Human rhinoviruses (HRVs) and enteroviruses (HEVs) belong to the *Enterovirus* genus in the *Picornaviridae* family. Despite high sequence homology and similar genomic organization [1], these viruses display different clinical presentations and pathogenesis. HRVs are highly prevalent in the human population and the cause of most common colds as well as complications, such as asthma/wheezing and chronic obstructive pulmonary disease exacerbations, acute otitis media, sinusitis, bronchitis, and lower respiratory tract diseases [2,3,4,5]. Infection is mostly restricted to the respiratory tract, but disseminated infections have also been described [6,7]. In contrast, HEVs are able to disseminate and have been associated with over 20 different diseases, including infections of the central nervous system [8].

HRVs are characterized by an elevated genetic diversity with more than 160 distinct types [9] grouped in three distinct species, HRV-A, HRV-B, and the recently described HRV-C species [10]. HRV-Cs are thought to cause more severe diseases with frequent influenza-like illnesses and extra-respiratory manifestations [6,7,11]. Similarly, HEVs count more than 100 types divided into four species, HEV-A to -D [9]. For this virus group, the genetic diversity is parallelled by an important phenotypic diversity since viruses from a given species can cause a variety of diseases from the common cold to poliomyelitis. There are some reports on the viral etiologies of respiratory infections [12,13,14] or HRV species distribution [15,16] among African children, but only one group studied HRV molecular epidemiology in Africa by comparing asymptomatic children with children presenting acute respiratory infection [17]. As a consequence, data regarding the full spectrum of HRV and HEV circulating strains are lacking, in part probably due to the absence of available diagnostic tools. The objective of this current work is to analyze HRV and HEV circulation and molecular epidemiology in respiratory specimens collected from febrile children in rural and urban areas of Tanzania.

## 2. Methods

Children enrolled in this investigation were part of a large prospective cohort assessing the causes of fever and respiratory disease in Tanzanian children [18]. Participants aged from two months to 10 years of age and presenting with an axillary temperature ≥38 °C were recruited in 1 urban and 1 rural hospital in Tanzania. Exclusion criteria were severe malnutrition and emergency signs according to World Health Organization criteria [19].

### 2.1. Microbiological Analysis

We collected naso- and oropharyngeal (NP/OP) swabs from all patients to test for respiratory viruses. Other diagnostic tests were performed as previously described [18]. NP/OP swabs were immediately placed in tubes containing Universal Transport Medium (Copan, Brescia, Italy). Nucleic acids were extracted with Easymag (bioMérieux, Geneva, Switzerland) according to the manufacturer’s instructions and either directly tested by real-time PCR for bocavirus and adenovirus, or reverse transcribed and screened by RT-PCR for influenza A, B and C, respiratory syncytial virus A and B, human metapneumovirus, parainfluenza virus 1 and 3, HRV, HEV, and coronavirus as described previously. Real-time PCR was performed using the QuantiTect Probe RT-PCR Kit (Qiagen, Hombrechtikon, Switzerland) according to the manufacturer’s instructions in an Applied Biosystems StepOne™ (Applied Biosystems, Foster City, CA, USA) [20,21]. Other microbiological analyses for non-respiratory viruses, bacteria, and parasites were performed as previously described [18].

### 2.2. PCR and Sequencing

Fragments of the viral genome were amplified on cDNA by PCR using the AmpliTaq polymerase (Applied Biosystems) or FastStart Taq DNA Polymerase (Roche Applied Sciences, Basel, Switzerland). 5' Untranslated region (UTR), VP4/VP2 and VP1 amplification and sequencing were performed with generic primers described in Table S1 [22,23,24], whereas degenerate and/or specific primers were designed to amplify and sequence further selected specimens (Table S2). Briefly, 390 nt of the 5'UTR were systematically PCR amplified with primers 11 and 23. For HRV-C and HEV, a VP4/VP2 semi nested PCR was also systematically attempted (primers 46/P1.204 and 47/P1.204 for HRV and 46/Ent_P1.39 and 47/Ent_P1.39). The same HRV specific semi-nested PCR was used to amplify the VP4/VP2 region of selected representatives of the different 5'UTR phylogenetic clusters found for HRV-A and -B. When enough specimens were available, specific primers were designed to amplify other genomic regions in case of unusual strains or inconsistent sequencing results (Table S2). In case of unsuccessful amplification, nested PCR was systematically attempted. PCR products were purified with MSB^®^ Spin PCRapace (Invitek, Hayward, CA, USA), sequenced with forward and reverse primers and analyzed by Geneious software 5.4.6 (Biomatters Ltd., Auckland, New Zealand). No PCR was attempted on samples with cycle threshold (CT) > 41. All sequences form recombinant or divergent strains as well as sequences of previously unavailable regions are deposited in GenBank under accessions KR997879 to KR997897 and KT751298 to KT751301.

### 2.3. Phylogenetic Analysis

Alignments were constructed using Muscle [25] with a maximum of 64 iterations. The 5'UTR, VP4/VP2 and VP1 regions included in the alignments for each species is described in the Supplementary Figures legend. HRV and HEV sequences from reference types were obtained from alignments published by Palmemberg *et al*. [26]. The sequences of HRV and HEV types identified after this publication were downloaded from [9].

The alignments were converted into PHYLogeny Inference Package (PHYLIP) format (for tree-building) with the EMBOSS program “seqret” [27]. Trees were built with PhyML [28] using the general time-reversible (GTR) and Bio Neighbor-joining (BIONJ) for the initial tree and optimized tree topology and branch lengths. Trees with fewer than 50 species used 16 rate categories and the larger trees used 8. Transition/transversion ratio, proportion of invariant sites, and the shape parameter (alpha) of the gamma distribution were estimated. Tree processing (including rooting and computation of support values) was performed with the Newick Utilities [29]. Tree images were created with FigTree [30].

### 2.4. Statistical Analyses

Statistics were performed using SPSS software (IBM SPSS Statistics for Macintosh, Version 22.0., IBM Corp, Armonk, NY, USA). For continuous variables, Student’s *t*-test or Mann-Whitney U-test were used to compare means or medians, depending on variable distribution. For categorical variables, chi-square test was used to compare groups.

### 2.5. Ethical Considerations

The procedures followed were in accordance with the ethical standards set by the Tanzanian and Swiss ethics committees and the Declaration of Helsinki as previously described [18]. The protocol was approved by the regional ethics committee in Basel, Switzerland, and by the national ethics committee in Tanzania. Written informed consent was obtained from the parents or guardians of each participant.

## 3. Results

**Figure 1:**
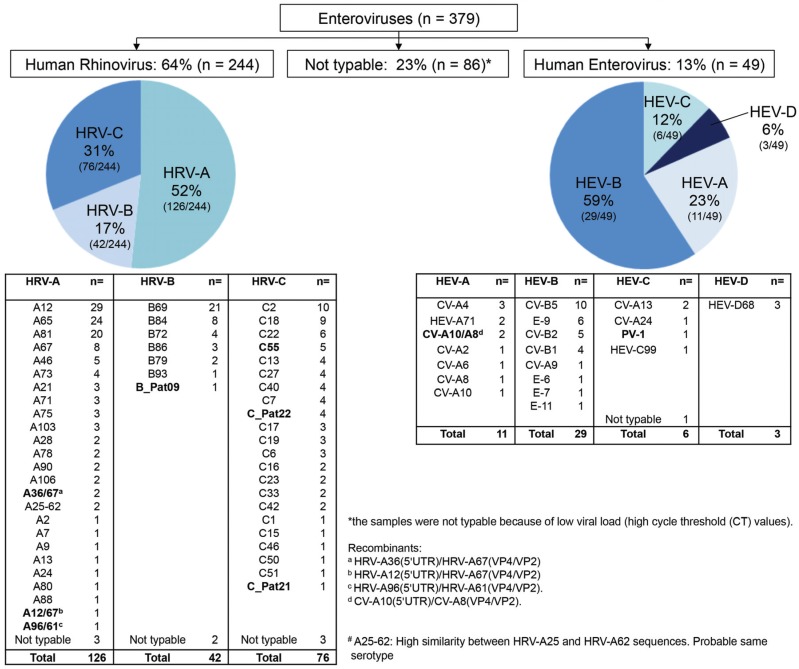
Distribution of human rhinoviruses (HRV) and enteroviruses (HEV) species and serotypes.

A total of 1005 NP/OP samples were collected from Tanzanian children <10 years old with an axillary temperature ≥38 °C from April through December 2008. Among these, 38% (378/1005) harbored an HRV or HEV infection; we highlighted one patient presenting a co-infection with two species (HRV-A67 and HEV-99). Twenty-three percent (86/379) of isolates were not typable because of high CT values and/or bad sample quality. Among the 293 typable *Enteroviruses* detected in 292 patients, 244 (83%) revealed to be HRV and 49 (17%) HEV (Figure 1). HRV-A types were the most frequent, followed by HRV-C and HRV-B. Among HEV, HEV-B were the most prevalent followed by HEV-A, -C and -D. Although some types were dominant, such as HRV-A12, -A65 and -A81, HRV-B69 and HRV-C2, and -C18 or CV-B5 for HEVs, many distinct types (54 distinct for HRV and 20 distinct for HEV) were co-circulating in our study population.

### 3.1. Associated Clinical and Epidemiological Features

**Figure 2:**
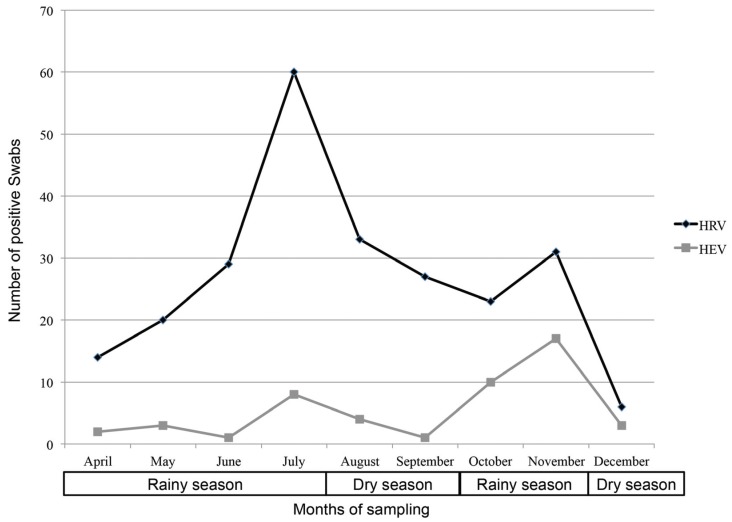
Repartition of positive swabs for human rhinoviruses (HRV) and enteroviruses (HEV) according to months of sampling.

Fifty-one percent of patients were female (148/292). Age was similar between HRV- (median [interquartile range (IQR)]: 13.9 months [8.8–25.2]) and HEV-infected (15.1 months [9.0–31.6]; *p* > 0.05) patients, as well as between those infected with HRV-A (14.1 months [7.5–24.8]), -B (17.1 months [10.1–25.1]) or -C species (12.3 months [8.9–28.6]; *p* > 0.05). Over the eight-month study period, HRV and HEV infection peaked from June to August (end of long rainy season/beginning of dry season) and from October to November (short rainy season), the maximum number of cases being from June to August for HRV, and October and November for HEV (Figure 2). HEV accounted for 11% of *Enteroviruses* infections in the rural hospital (12/110), compared to 20% in the urban hospital (37/182; *p* > 0.05).

**Figure 3:**
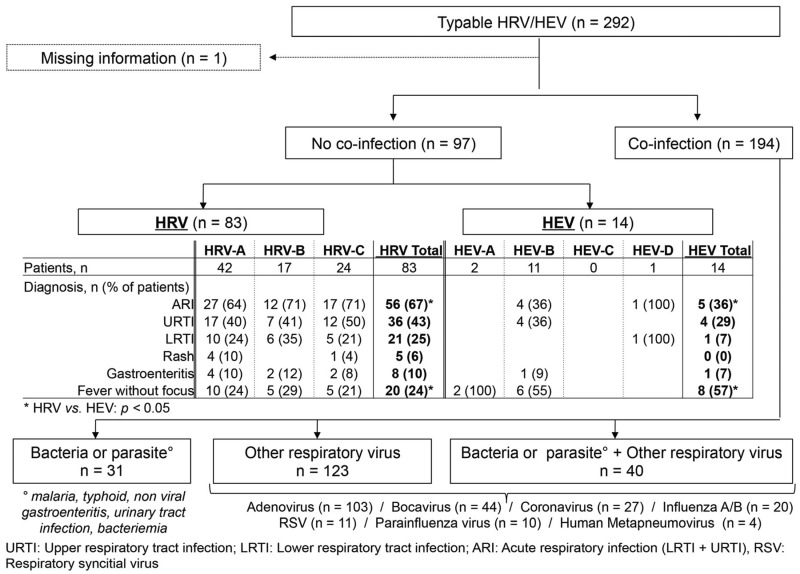
Flowchart of typable human rhinoviruses (HRV) and enteroviruses (HEV) according to presence of co-infection and diagnosis.

Co-infection with at least one other agent was documented in 194/292 (66%) patients. Thirty-one were co-infected with a bacteria/parasite (malaria, typhoid, non-viral gastroenteritis, urinary tract infection, and bacteremia), 123 with another respiratory virus (influenza, respiratory syncytial virus, human metapneumovirus, parainfluenza virus, coronavirus, bocavirus, adenovirus) and 40 with a bacteria/parasite plus a respiratory virus (Figure 3). The proportion with viral respiratory co-infection was similar between HEV- (29/49; 59%) and HRV-infected patients (134/244; 55%; *p* > 0.05). The most frequently identified respiratory viruses were adenovirus (*n* = 103 patients), bocavirus (*n* = 44), coronavirus (*n* = 27), and influenza virus (*n* = 20). Prevalence of respiratory virus co-infection was not statistically different between HRV-A (70/126; 56%), -B (23/42; 55%) and -C (41/75; 55%; *p* > 0.05) species.

In mono-infected cases, CT values were lower for HRV than for HEV (mean CT values ± standard deviation [SD] 31.1 ± 4.6 *vs.* 34.6 ± 5.0; *p* < 0.05). Mean CT values were not statistically different between HRV-A (30.6 ± 3.8), -B (31.7 ± 4.6) and -C (31.7 ± 5.9; *p* > 0.05), as well as between the different HEV types. After exclusion of patients in whom another possible cause of fever was identified, there remained 83 patients infected only with HRV and 14 infected only with HEV (Figure 3). For HRV, the most frequent diagnoses were upper respiratory tract infection (URTI), followed by lower respiratory tract infection (LRTI) and, surprisingly, gastroenteritis and rashes. HRV-C was not associated with a more severe presentation compared to other HRV species. For HEV, URTI was predominant, followed by LRTI and gastroenteritis.

Acute respiratory infection, which includes URTI and LRTI, was more prevalent among HRV- (56/83) than HEV-infected patients (5/14; *p* < 0.05). By contrast, fever without focus was more prevalent among HEV- (8/14) than among HRV-infected patients (20/83; *p* < 0.05). The proportion of patients presenting respiratory symptoms, headache, runny nose, cough, throat pain, vomiting, diarrhea, abdominal pain, back pain, ear pain, fever ≥ 39 °C, tachypnea, dehydratation, red pharynx, swollen or red tonsils, mouth lesions (white, ulcer), rashes, chest indrawing, noseflap, stridor, grunting, abnormal chest ausculation, wheezing, abnormal abdominal palpation, adenopathy, or severe infection was not statistically different between HRV- and HEV-infected patients, irrespective of whether patients with documented co-infection were excluded or not.

### 3.2. Molecular Epidemiology of HRV-A (n = 126)

The most frequently identified types were HRV-A12 (*n* = 29), HRV-A65 (*n* = 24), and HRV-A81 (*n* = 20) (Table S3 and Figure S1A [5'UTR tree], S1B [VP4/VP2 tree]). Of note, sequences that did not segregate properly in the 5'UTR or VP4/VP2 trees were genotyped based on BLAST results (Table S3). Several contemporary recombinants were identified among circulating HRV-A strains with three involving HRV-A67. Two patients (918T and 976T) harbored a recombinant virus between HRV-A36 (5'UTR) and HRV-A67 (VP4/VP2) while another strain (from patient 91T) was a recombinant between HRV-A12 (5'UTR) and HRV-A67 (VP4/VP2) (Figure S1A [5'UTR tree], S1B [VP4/VP2 tree] and Table S3). Of note, several classical non-recombinant HRV-A12 and HRV-A67 strains co-circulated in the same population. Another recombinant between HRV-A96 (5'UTR) and HRV-A61 (VP4/VP2) was also identified in patient 265T (Figure S1A [5'UTR tree], S1B [VP4/VP2 tree] and Table S3). Interestingly, one strain clustered with HRV-A62 on 5'UTR and HRV-A25 on VP4/VP2, whereas another strain clustered with HRV-A25 on 5'UTR and HRV-A62 on VP4/VP2. However, these strains were not considered as recombinants as HRV-A25 and HRV-A62 presented 11% nt divergence over VP1 and, based on the proposed threshold for type assignment [31], should be considered as a unique HRV-A type. One patient presented a co-infection between HRV-A67 and HEV-C99.

### 3.3. Molecular Epidemiology of HRV-B (n = 42)

The most frequent type was HRV-B69 (*n* = 21), followed by HRV-B84 (*n* = 8) and HRV-B72 (*n* = 4) (Table S4 and Figure S2A [5'UTR tree], S2B [VP4/VP2 tree]). A divergent HRV-B strain (patient 736T), with an 87% nt identity on 5'UTR with HRV-B72 (GenBank entry: JN798562.2), 85% on VP4/VP2 with HRV-B27 (GenBank entry: FJ445186.1), and 78% on VP2/VP3 with HRV-B84 (GenBank entry: JQ837723.1) was identified and provisionally assigned as “HRV-B_Pat09” due to the lack of VP1 sequence (Figure S2A [5'UTR tree], S2B [VP4/VP2 tree] and Table S4) [32]. Four highly related strains at the VP4/VP2 level were also identified in Cambodia and China (GenBank entries: KF034021.1, KF879892.1). No recombinant was highlighted among HRV-B viruses.

### 3.4. Molecular Epidemiology of HRV-C (n = 76)

Among all HRVs, 31% (76/44) were HRV-C (Figure 1). The most frequent were HRV-C2 (*n* = 10), HRV-C18 (*n* = 9), and HRV-C22 (*n* = 6) (Table S5 and Figure S3A [5'UTR tree], S3B [VP4/VP2 tree]). Ten patients harbored divergent viral strains. One strain (patient 529T) presented a 92% identity on 5'UTR with HRV-C35 (GenBank entry: JF436925.1) but was highly divergent on VP4/VP2 (80% with HRV-C35; GenBank entry: EU081790.1). Longer sequences such as 5'UTR/2A presented an 81% nt identity with HRV-C35 (GenBank entry: JF436925.1), thus confirming a new type (assigned as “HRV-C55”). Four other patients (583T, 584T, 626T, 668T) harbored viruses that clustered with this strain on the 5'UTR and, when available VP4/VP2, suggesting that they all belonged to this new HRV-C55 (Table S5). Of note, several strains highly related on VP4/VP2, but unassigned, have been also identified, particularly in Jordan, the Philippines, and China (GenBank entries: FJ615748.1, FJ615747.1, AB683948.1, GQ223148.1 and JF316844.1). Similarly, another strain (624T) was highly divergent with 81% nt identity on 5'UTR with HRV-C54 (GenBank entry: KP282614.1), 85% on VP4/VP2 with HRV-C48 (GenBank entry: JF519763), and 74% on VP2/VP3 with HRV-C28 (GenBank entry: JN798569.1) (Table S5). However, this strain clustered with the provisionally assigned HRV-C_Pat22 type (GenBank entry: FJ615745) [31,33]. Two other patients (617T and 644T) harbored the same sequences. Furthermore, because of its highly similar 5'UTR sequence (>99.1% identity), we assume that a fourth patient (710T) also carried this new HRV-C_Pat22. A last strain (813T) was relatively conserved on 5'UTR (91% with HRV-C36; GenBank entry: KF499421.1), but was below the threshold for new type assignment with 85% nt identity on VP4/VP2 with HRV-C36 (GenBank entry: KF688657.1) and 84% on 5'UTR/VP3 with HRV-C36 (GenBank entry: JN541267.1) (Table S5). This strain clustered with the provisionally assigned HRV-C_Pat21 type (GenBank entry: FJ615737) [31,33]. Of note, for these divergent HRV-C strains, VP1 could not be sequenced due to high CT values and probable mismatches with designed primers.

Finally, our 5'UTR sequences complete the available dataset for HRV-C13, -C16, -C18, -C19, -C23, -C27, -C33, and -C46, as well as the VP3 sequence for HRV-C23 (Table S5). Due to an important lack of available typed HRV-C 5'UTR, 5'UTR HRV-C recombinants could not be highlighted in this study.

### 3.5. Molecular Epidemiology of HEV

Forty-nine HEVs were identified. Proportions of HEV-A, HEV-B, HEV-C and HEV-D were 23% (11/49), 59% (29/49), 12% (6/49), and 6% (3/49), respectively (Figure 1).

Among HEV-A, the most frequently identified types were coxsackievirus (CV)-A4 (*n* = 3) and HEV-A71 (*n* = 2) (Table S6 and Figure S4A [5'UTR tree], S4B [VP4/VP2 tree] and S4C [VP1 tree]). Two patients (48T and 477T) harbored a virus presenting incongruity between the 5'UTR (clustering with CV-A10) and the VP4/VP2 (clustering with CV-A8) phylogenetic trees (Figures S4D and S4E). CV-A10 and CV-A8 were also identified in the cohort, thus confirming co-circulation. None of the two patients with HEV-A71 infection presented severe or neurological symptoms.

Among HEV-B, the most frequent types were CV-B5 (*n* = 10), E-9 (*n* = 6) and CV-B2 (*n* = 5) (Table S7 and Figure S5A (5'UTR tree), S5B (VP4/VP2 tree) and S5C (VP1 tree)).

Among HEV-C, CV-A13 were predominant (Table S8 and Figures S6A [5'UTR tree], S6B [VP4/VP2 tree] and S6C [VP1 tree]). We identified a nine-month-old patient (593T) harboring vaccinal poliovirus type 1 (PV1) based on 5'UTR, VP4/VP2, VP3/VP1, 2C, and 3A/3C typing in NP/OP secretions (Table S8). The overall nt difference with the Sabin vaccine was of 0.54% in the P1 region, 0.7% in the 5'UTR, and 0.52% in 3D. The child presented with a runny nose and vomiting, but no neurological symptoms. The definitive diagnosis was acute otitis media and typhoid fever. No other respiratory pathogen was present in the NP/OP sample, suggesting that this virus was responsible for the respiratory symptoms. The vaccine history was unfortunately not available. Viral inoculation in cell culture could not be performed due to limited clinical material and high CT value (33.3).

Among HEV-D, all patients were infected with HEV-D68 (Table S9 and Figure S7 [5'UTR/VP2 tree]). Two presented respiratory symptoms (pneumonia and obstructive bronchitis, respectively), but none presented severe or neurological symptoms. It is also important to note that two patients were also co-infected with other respiratory viruses (one with parainfluenza virus and one with coronavirus and adenovirus).

## 4. Discussion

*Enteroviruses* are the most frequent cause of infections worldwide, but data are sparse from the African region. This study presents an overview of the molecular epidemiology of HRV and HEV in respiratory samples from children with fever in Tanzania. Globally, the proportion of patients with a positive NP/OP swab for HRV or HEV was higher than expected. Even more interesting was the minority of patients with a mono-infection. Indeed, only one-third of HRV- or HEV-infected individuals were infected solely with these viruses. After exclusion of patients with documented co-infection, respiratory infections were as expected the most frequent diagnoses in HRV-infected individuals, but rashes and gastroenteritis were surprisingly high. Since HRVs do not replicate in the gastro-intestinal tract due to sensitivity to acidic pH, this may be linked to underlying host conditions or non-documented co-infection rather than viral infection. HRV-C was not associated with a more severe presentation compared to other HRV species. Notably, no severe respiratory symptoms were linked to HRV-B and the CT values were higher in this group, corroborating the idea that HRV-B specie is less pathogenic than HRV-A or -C [34]. For HEV-infected patients, respiratory infections such as LRTI and URTI accounted for the most frequent diagnoses attributable to HEV. This is in line with the report of Jacques *et al*., which showed that respiratory infection was the second most frequent clinical entity due to HEV [35]. We observed a relatively high prevalence of HEV in NP/OP secretions: 17% of patients infected with a typable *Enterovirus* were carrying an HEV. Given that our screening assay is slightly less sensitive on HEV than on HRV, this represents a true finding and not a screening bias [21]. The high proportion of HEV in respiratory samples is concordant with previous epidemiological surveillance conducted in South Africa, New Caledonia and Cameroon [4,14,36], whereas studies in Switzerland, Senegal and Mozambique showed a lower proportion of HEV [12,13,20]. However, HEV CT values were high, thus suggesting that their presence in NP/OP swabs may have been the consequence of a disseminated HEV infection predominating in another organ, such as the digestive tract. The lower sensitivity of the screening assay on HEV than on HRV may also partially explain why CT values were higher in HEV compared to HRV.

The high rate of viral co-infections could be explained by simultaneous or sequential infections with prolonged viral shedding, as well as asymptomatic carriage of one or more viruses. For example, HRV can be found in the nasopharynx of up to 40% of children without active symptoms [37,38,39,40], especially among the very young [37]. Molecular techniques have led to the characterization of new clinically relevant pathogens and have improved diagnostic sensitivity. As HRV infection prevalence has been shown to be correlated with humidity [41], it is also possible that the proportion of patients with HRV in their nasopharynx is higher in Tanzania than in temperate countries. It explains also the peaks of HRV infection during rainy seasons. In addition, recruitment was not performed during a full year, but from April to December, and this may have provided more information concerning HRV and HEV seasonality and distribution.

Interestingly, our phylogenetic analysis highlighted divergent and recombinant strains, with four divergent HRV strains (HRV-B_Pat09, HRV-C55, HRV-C_Pat 21 and HRV-C_Pat22), as well as intraspecies HEV-A and HRV-A recombinants. Reports about contemporary HRV recombinants are rare and always linked to HRV-A intraspecies recombinants [31,42]. Among the identified recombinants, the HRV-A36/A67 recombinant clusters with previously documented similar recombinants (GenBank entries: EU840918.1, EU840872.1, EU840750.1, EU840930.1, EU840884.1 and EU840762.1) identified in Switzerland and indicates a worldwide circulation [42]. Interestingly, another HRV-A67 recombinant (HRV-A12/A67) was identified with the same recombination breakpoint on HRV-A67. Thus, HRV-A67 5'UTR may represent a recombination hotspot. Moreover, parental strains, such as CV-A8, CV-A10, HRV-A12 and HRV-A67, were identified in our cohort, thus confirming co-circulation with CV-A10/CV-A8, HRV-A12/HRV-A67 and HRV-A36/HRV-A67 recombinants. This indicates that recombinant strains did not outcompete parental strains among circulating viruses. We achieved 5'UTR sequences of many HRV-C types, as well as the VP3 sequence of HRV-C23, which allowed to complete the databases currently available for these viruses [9]. This may help to identify potential HRV-C 5'UTR recombinants in future epidemiology studies.

Genotyping also revealed that more than 50 different HRV types circulated in the same community during the same season as observed in previous studies [42,43]. In addition, the majority of strains identified in Tanzania did not differ significantly from those circulating in other continents and suggests wordwide distribution of the same viruses, independent of environmental conditions. Many different HEV types were identified in our cohort, including HEV-D68 and HEV-A71, as well as vaccine strains, such as PV1. However, HEV type distribution was unexpected as most of the types identified are usually transmitted via the fecal-oral route (as is the polio vaccine). Moreover, a significant proportion (CV-A8, CV-A13, PV-1 and HEV-C99) is usually not found in respiratory samples [8,35]. This may be explained by suboptimal hygiene predisposing to fecal-oral transmission or may indicate that the children’s immune system, together with potential micronutrient deficiency, could contribute to HEV infection and increase the proportion of disseminated disease. The patient presenting with PV1 carriage in a NP swab presented with URTI and vomiting, but no neurological complaints. Nasal carriage could be due to a recent direct immunization with oral polio vaccine or passive transmission of the virus from another vaccinated child; however, the incomplete vaccinal history cannot confirm this. Nevertheless, the presence of PV vaccine in the respiratory sample suggests that passive immunization could occur also via the respiratory route and should be taken into account in the polio eradication program. HEV-D68 and HEV-A71 present the ability to reach the central nervous system (CNS) and cause complicated disease as shown in the recent outbreak in the USA for HEV-D68 [44] or in HEV-A71 [45] epidemics in Asia Pacific countries. We identified two patients with HEV-A71 and three patients with HEV-D68 infections but none of them presented complicated disease.

HRV-A and HRV-C were more prevalent than HRV-B, an observation that is in line with other studies in Africa, Europe, and the USA [3,15,16,17,22,38,46]. The relative prevalence of HRV-A in our study is also similar to other reports worldwide [3,15,38,46], although some African and Asian pediatric studies showed a higher or similar HRV-C prevalence compared to HRV-A [16,17,22].

## 5. Conclusions

This is the first prospective study focused on HRV and HEV molecular epidemiology in sub-Saharan Africa. The systematic and carefully conducted screening of more than 1000 febrile children provides important clinical information, highlights primordial features about HRV and HEV evolution and circulation, and confirms the wide genomic diversity of these species. HRV-A intraspecies recombinant highlights HRV natural evolution, whereas new putative HRV-B and HRV-C types warrant other large molecular epidemiologic studies outside Europe and the USA to complete the list of circulating *Enterovirus* types.

## Supplementary Materials

The following are available online at www.mdpi.com/1999-4915/7/12/2948/s1. Figure S1A: HRV-A 5'UTR tree (nt 240 to 449 relative to HRV-A2_X02316), Figure S1B: HRV-A VP4/VP2 tree (nt 611 to 1030 relative to HRV-A2_X02316), Figure S2A: HRV-B 5'UTR tree (nt 177 to 449 relative to HRV-A2_X02316), Figure S2B: HRV-B VP4/VP2 tree (nt 611 to 1018 relative to HRV-A2_X02316), Figure S3A: HRV-C 5'UTR tree (nt 240 to 449 relative to HRV-A2_X02316), Figure S3B: HRV-C VP4/VP2 tree (nt 611 to 1075 relative to HRV-A2_X02316), Figure S4A: HEV-A 5'UTR tree (nt 177 to 370 relative to HRV-A2_X02316), Figure S4B: HEV-A VP4/VP2 tree (nt 611 to 1030 relative to HRV-A2_X02316), Figure S4C: HEV-A VP1 tree (nt 2429 to 2709 relative to HRV-A2_X02316), Figure S4D: HEV-A 5'UTR recombinant tree (nt 169 to 611 relative to HRV-A2_X02316), Figure S4E: HEV-A VP4/VP2 recombinant tree (nt 611 to 1038 relative to HRV-A2_X02316), Figure S5A: HEV-B 5'UTR tree (nt 193 to 611 relative to HRV-A2_X02316), Figure S5B: HEV-B VP4/VP2 tree (nt 661 to 993 relative to HRV-A2_X02316), Figure S5C: HEV-B VP1 tree (nt 2429 to 2705 relative to HRV-A2_X02316), Figure S6A: HEV-C 5'UTR tree (nt 281 to 557 relative to HRV-A2_X02316), Figure S6B: HEV-C VP4/VP2 tree (nt 611 to 934 relative to HRV-A2_X02316), Figure S6C: HEV-C VP1 tree (nt 2450 to 2743 relative to HRV-A2_X02316), Figure S7: HEV-D 5'UTR/VP2 tree (nt 661 to 993 relative to HRV-A2_X02316). Table S1: Generic primer used for initial screening, Table S2: Primers used to sequence selected specimens, Table S3: HRV-A genotyping according to sequenced genome regions, Table S4: HRV-B genotyping according to sequenced genome regions, Table S5: HRV-C genotyping according to sequenced genome regions, Table S6: HEV-A genotyping according to sequenced genome regions, Table S7: HEV-B genotyping according to sequenced genome regions, Table S8: HEV-C genotyping according to sequenced genome regions, Table S9: HEV-D genotyping according to sequenced genome regions.
