# Supplementary material for: Molecular Epidemiology of Human Rhinoviruses and Enteroviruses Highlights Their Diversity in Sub-Saharan Africa
Source: Viruses. 2015 Dec 8;7(12):6412–23. doi: 10.3390/v7122948 (PMC4690871; doi:10.3390/v7122948)
Supplement: Supplementary file 1 [file viruses-07-02948-s001.pdf]

Supplementary Materials: Molecular Epidemiology of Human Rhinoviruses and Enteroviruses Highlights Their Diversity in Sub-Saharan Africa

Arnaud G. L'Huillier, Laurent Kaiser, Tom J. Petty, Mary Kilowoko, Esther Kyungu, Philipina Hongoa, Gaël Vieille, Lara Turin, Blaise Genton, Valérie D'Acremont and Caroline Tapparel

Table S1. Generic primer used for initial screening.

Target	Primer ID	Gene:	Name	Sequence	Position *	Sense	Reference
all HRV/all HEV	11	5'UTR	HRVfor	GCACTTCTGTTTCCCC	164	F	[1]
all HRV/all HEV	23	5'UTR	RevA_B 550Rhino	GAAACACGGACACCCAAAGTAGT	554	R	[1]
all HRV/all HEV	46	5'UTR	F484	CGGCCCCTGAATGYGGCTAA	484	F	[2]
all HRV	P1.2	VP2	VP2Fwd1049-deg	TGGTGSTGGAARYTRCCWGATGC	~1049	F	[1]
all HRV	P1.204	VP2	R1126	ATCHGGHARYTTCCAMCACCA	1126	R	[2]
all HEV	Ent_P1.39	VP2	R1126ENT	GGDAAYTTCCACCACCAHCC	1126	R	This study
all HEV	Ent_P1.16	VP3	224	GCIATGYTIGGIACICAYRT	1977–1996PV1	F	[3]
all HEV	Ent_P1.15	VP1	222	CICCIGGIGGIAYRWACAT	2969–2951PV1	R	[4]
all HEV	Ent_P1.17	VP1	AN89	CCAGCACTGACAGCAGYNGARAYNGG	2602–2627	F	[3]
all HEV	Ent_P1.18	VP1	AN88	TACTGGACCACCTGGNGGNAYRWACAT	2977–2951	R	[3]

* Postitions are indicated relative to HRV-A2 (NCBI GenBank entry: X02316).

Table S2. Primers used to sequence selected specimens.

Target Sample	N°oligos	Gene	Name	Sequence	Position *	R/F
671/918/949/976/984	1.54	VP2	VP2fwd 1334 HRV A	TGGCTHAATTKTGATGGHAC	1334 to 1353	F
HRV-C	P1.223	VP2	VP2Fwd HRVC	GATAGGCTMAARCAAATCACTAT	848 to 870	F
HRV-C	P1.224	VP2	VP2Fwd HRVCn	GCWTATGGDGAATGGCC	917 to 933	F
671T-984T-949T	P1.233	VP2	VP2Fwd HRVA	GGWGTTGGCCACAATATC	924 to 941	F
671T-984T-949T	P1.234	VP2	VP2Fwd HRVAn	GATGCTGATGCMAATGC	947 to 963	F
344T-671T	P1.237	VP2	VP2Fwd HRVA	ATATGGAGTCTGGCCACA	919 to 936	F
344T-671T	P1.238	VP2	VP2Fwd HRVAn	CTATAGATAAACCATCACACCC	1186 to 1207	F
529T	P1.252	VP2	VP2Fwd529T-1525	CCCACTAAACTCATACACATCCC	1668 to 1710	F
344T	P1.256	VP2	VP2Fwd344T_71p1044-1319	GCACACTCATCCAGGTGAAAC	1252 to 1272	F
344T	P1.257	VP2	VP2Fwd344T71_p1044-1377	GACAGGGCAGCCGACAG	1310 to 1325	F
344T	P1.258	VP2	VP2Rev344T71_p1044-1480	CATAGGGTAGAATGAGTGTGGCTG	1436 to 1413	R
344T	P1.259	VP2	VP2Rev344T71_p1044-1564	CAGCTGTAAAGGGGAGATGG	1511 to 1496	R
543T	P1.263	VP2	VP2Rev543T-1088	GATCCGGCTCCTATATAAGCC	1233 to 1217	R
639T-1379	P1.268	VP2	VP2Fwd639T-1379	CTCCCACAGTGTCAATAACCG	1530 to 1547	F
701T-1450	P1.270	VP2	VP2Fwd701T-1450	GTCAACCGATGGATAACATGC	1695 to 1715	F
918T	P1.276	VP2	VP2-1054-918T_Fwd	GGAGAGCAAGACTGGAGTGC	1015 to 1035	F
122T	P1.280	VP2	VP2-1113-122T Fwd	CACTATCTTCCTCACCAAATATAACC	1369 to1397	F
610T	P1.285	VP2	VP2-1398-610T Fwd	CCTATAAGTCTGAATCTCTGGCGC	1546 to 1584	F
882T	P1.288	VP2	VP2-850-882T Fwd	GATAGGCTCAAGCAAATACTAT	848 to 868	F
all HEV	Ent_P1.63	VP2	VP2 Fwd HEV	CCATCACCACTCAAGARGC	882 to 900	F

Table S2. *Cont.*

Target Sample	N°oligos	Gene	Name	Sequence	Position *	R/F
all HEV	Ent_P1.64	VP2	VP2 Fwd HEVn	CCGTGGACAARCCWAC	963 to 978	F
288/639/671/918	P1.15	VP3	Primer HRVA C1	ACNTGBTGGTAYCARAC	2165 to 2181	F
529T	P1.253	VP3	Vp3Rev529T-1902	GATGATGCAGGCCTAGATCC	2090 to 2065	R
543T-1747	P1.264	VP3	VP3Fwd543T-1747	GCATACCCCTCCAGATGG	1858 to 1876	F
701T-C17_C28_N4T-2265	P1.271	VP3	Vp3Rev701T-C17_C28_N4-2265	AACATCTRACDGABARATCWGG	2270 to 2248	R
987T-1718	P1.272	VP3	Vp3Fwd987T-1718	CCTGAGTATAGTCCTACAAAGGAG	1677 to 1697	F
918T	P1.277	VP3	P1.277 Vp3-2363-918T Rev	CTGGGTTGGGTAATGGG	2318 to 2300	R
976T	P1.278	VP3	P1.278 VP3-2345-976T Fwd	GCTCTGTTGTATCTGCATGMAA	2226 to 2247	F
976T	P1.279	VP3	P1.279 VP3-3249-976T Rev	AGATTCCCACATGCACAT	3205 to 3187	R
882T	P1.290	VP3	P1.290 VP3-1606-882T Fwd	CAGGGAATCCCAGTGCGC	1600 to 1615	F
590/648/948	P1.9	VP1	Primerhovi_PRPP	TGGTGYCCIMGISCICCIMGTGC	~3044 to 3066	F
671/918	1.19	VP1	HRVA Primer 2	YCCWCCACARTCWCCWGGTTC	3484 to 3464	R
288/639	P1.20	VP1	HRVA Primer 3	HATRCCWATVACWCCATG	3517 to 3500	R
265	1.64	VP1	Vp1 rev 2412-A	GTRTGYCCTGTYTCAGCAGCATCYA	2436 to 2412	R
HRV-C	P1.160	VP1	HRVC VP1-1922 Fwd	TRGCMTACACMCCHCCAGG	1992 to 2010	F
HRV-C	P1.161	VP1	HRVC VP1-2190 Fwdn	GCATGHTRCGTGAYACACCMATGAT	2262 to 2286	F
529T-539T-543T-566T-624T-813T-882T-987T	P1.225	VP1	VP1RevHRVCextr1	GWCGDGGRATCCATGC	3056 to 3041	R
529T-539T-543T-566T-624T-813T-882T-987T	P1.226	VP1	VP1RevHRVCextr1n	GTYCCCATGTCATTGT	1089 to 1074	R
122T-288T-383T-386T-610T-638T-701T	P1.228	VP1	VP1RevHRVCextr2 m	ACAWACATGATYTGCATAA	2748 to 2730	R
671T-984T-949T	P1.236	VP1	VP1Rev HRVAn	TGCCAAAAGACTGATGC	2826 to 2810	R

Table S2. *Cont.*

Target Sample	N°oligos	Gene	Name	Sequence	Position *	R/F
344T-671T	P1.239	VP1	VP1Rev HRVA	CATCATAGAACATGTAATATGCTGA	2900 to 2876	R
344T-671T	P1.240	VP1	VP1Rev HRVAn	AAAAGACTGAWGCATAGTCC	1786 to 1763	R
671T	P1.241	VP1	671_VP1Rev	CCTGGTATAAATATYTCTTGTG	1995 to 1974	R
383T_387T	P1.244	VP1	383_387VP1Rev	ATCYTCAGTGGTCATAAAYTGTG	1936 to 1914	R
529T_624T_566T_543T_987T_813T	P1.246	VP1	529_624_566_543_987_813VP1Rev	CCWGGRGGKGTGTATGCTA	2010 to 1992	R
539T	P1.248	VP1	539_VP1Rev	GGCCTGTGTGTGACCG	2599 to 2584	R
529T	P1.254	VP1	Vp1Fwd529T-2431	CAGGAGCAAGCACAAATCAG	2615 to 2634	F
529T	P1.255	VP1	Vp1Rev529TsubC35-3117	CAACCCTCCATGCAATCAC	3294 to 3276	R
344T	P1.260	VP1	Vp1Rev344T71_p1044-2527	CATGCTGGGTGGATATTG	2373 to 2354	R
344T	P1.261	VP1	Vp1Rev344T71_p1044-2458	GGGTCTGTCAATGTGACTG	2306 to 2285	R
122T	P1.262	VP1	VP1Rev122T-2336	CAGAAGCTTGAGACCAGGTG	2771 to 2751	R
T543TC02_p1009T-2717	P1.265	VP1	VP1Rev543TC02_p1009-2717	GTAGTATGCTGAACCTAGGCCAG	2806 to 2828	R
T639T-2323	P1.269	VP1	VP1Rev639T-2323	ATGTGTGCCTGTCCTGGAAG	2600 to 2580	R
Vp987T-C02_LZ508T-2468	P1.273	VP1	VpRev987T-C02_LZ508-2468	GCMCCAATTCCATMGCA	2424 to 2407	R
344T	P1.274	VP1	P1.274 VP1-2215_344T_Fwd	GACAAACTTGTGGTCCCCC	2169 to 2189	F
344T	P1.275	VP1	P1.275 VP1-3403_344T_Rev	TGCAATCACAAGTGGGGATG	3323 to 3304	R
610T	P1.286	VP1	P1.286 Vp1-2444-610T Fwd	CCTAGGAAGGTCAGCCTGTG	2514 to 2534	F
610T	P1.287	VP1	P1.287 Vp1-3350-610T Rev	CCCACAATCCCCTGGCTC	3481 to 3464	R
882T	P1.289	VP1	P1.289 Vp2-1743-882T Rev	GAGTCCACCCGTGCCATG	1752 to 1735	R
882T	P1.291	VP1	P1.291 Vp1-2907-882T Rev	GTGCTCTAATACATAGGTGGCCC	2950 to 2928	R

Table S2. *Cont.*

Target Sample	N°oligos	Gene	Name	Sequence	Position *	R/F
all HEV	Ent_P1.65	VP1	VP1 Rev HEV	TAMCCRTCRTAGAACCAYTG	2908 to 2885	R
all HEV	Ent_P1.66	VP1	VP1 Rev HEVn	GGGTKGTNGCWGTYTGCCA	2728 to 2710	R
590/648/948	EntP2.11	2C	COXA 19 4450 rev	TCAATRCGGTGTTTGSTCTTGAACTG	4846 to 4821	R
383	P2.64	2A	QPM-3098-REV	GGTATGTTGTCWGGSCCWGG	3309 to 3290	R

* Postitions are indicated relative to HRV-A2 (NCBI GenBank entry: X02316).

Table S3. HRV-A genotyping according to sequenced genome regions.

Patient ID	CT Value	Genotype	5'UTR	VP4/VP2	other
129	37.93	HRV-A NT	HRV-A NT		
508	35.13	HRV-A NT	HRV-A NT		
929	33.59	HRV-A NT	HRV-A NT		
485	37.27	HRV-A2	HRV-A2[a]	HRV-A2	
455	25.99	HRV-A7	HRV-A7	HRV-A7	
497	26.69	HRV-A9	HRV-A9	HRV-A9[b]	
26	29.55	HRV-A12		HRV-A12	
49	35.36	HRV-A12	HRV-A12	HRV-A12	
62	35.04	HRV-A12	HRV-A12	HRV-A12	
81	29.23	HRV-A12	HRV-A12	HRV-A12	
85	29.76	HRV-A12	HRV-A12		
89	30.65	HRV-A12	HRV-A12	HRV-A12	

Table S3. *Cont*.

Patient ID	CT Value	Genotype	5'UTR	VP4/VP2	other
148	37.88	HRV-A12	HRV-A12		
188	29.02	HRV-A12	HRV-A12	HRV-A12	
208	34.9	HRV-A12	HRV-A12	HRV-A12	
269	29.59	HRV-A12		HRV-A12	
306	25.54	HRV-A12	HRV-A12	HRV-A12	
369	27.18	HRV-A12	HRV-A12		
393	22.81	HRV-A12	HRV-A12		
400	31.03	HRV-A12	HRV-A12		
451	39.32	HRV-A12	HRV-A12		
476	32.97	HRV-A12	HRV-A12		
495	34.21	HRV-A12	HRV-A12	HRV-A12	
517	26.1	HRV-A12	HRV-A12		
532	31.78	HRV-A12	HRV-A12		
536	24.69	HRV-A12	HRV-A12		
541	26.7	HRV-A12	HRV-A12		
544	31.97	HRV-A12	HRV-A12		
561	31.81	HRV-A12	HRV-A12		
564	29.62	HRV-A12	HRV-A12		
578	30.59	HRV-A12	HRV-A12		

Table 3. *Cont.*

Patient ID	CT Value	Genotype	5'UTR	VP4/VP2	other
661	34.15	HRV-A12	HRV-A12	HRVA-12	
791	30.45	HRV-A12	HRV-A12		
795	38.51	HRV-A12	HRV-A12		
802	29.14	HRV-A12	HRV-A12 [c]		
91	28.86	HRV-A12/67Access nr: KT751299	HRV-A12	HRV-A67	
963	35.86	HRV-A13	HRV-A13	HRV-A13	
187	25.62	HRV-A21	NT	HRV-A21	
980	28.16	HRV-A21	NT	HRV-A21	
999	32.72	HRV-A21	HRV-A21	HRV-A21	
937	31.56	HRV-A24	HRV-A104	HRV-A24	
752	30.63	HRV-A25-62	HRV-A25-62	HRV-A25-62	
983	34.6	HRV-A25-62	HRV-A25-62	HRV-A25-62	
348	34.07	HRV-A28	HRV-A28	HRV-A28	
531	30.43	HRV-A28	HRV-A28	HRV-A28	
918	23.96	HRV-A36/67Access nr: KT751301	HRV-A36	HRV-A67	VP1: HRV-A67
976	33.99	HRV-A36/67Access nr: KT751300	HRV-A36	HRV-A67	VP1: HRV-A67
82	35.92	HRV-A46	HRV-A46	HRV-A46	
93	29.83	HRV-A46	HRV-A46		
318	33	HRV-A46	HRV-A46		

Table 3. *Cont.*

Patient ID	CT Value	Genotype	5'UTR	VP4/VP2	other
538	32.96	HRV-A46	HRV-A46		
551	29.8	HRV-A46	HRV-A46		
97	31.74	HRV-A65	HRV-A65-A102 [d]	HRV-A65	
123	32.07	HRV-A65	HRV-A65-A102 [d]	HRV-A65	
212	31.95	HRV-A65	HRV-A65-A102 [d]	HRV-A65	
239	33.71	HRV-A65	HRV-A65-A102 [d]	HRV-A65	
304	34.24	HRV-A65	HRV-A65-A102 [d]	HRV-A65	
327	30.84	HRV-A65	HRV-A65-A102 [d]	HRV-A65	
968	35.87	HRV-A65 [e]	HRV-A65-A102 [d]	HRV-A65	
272	32.37	HRV-A65		HRV-A65	
335	27.19	HRV-A65 [e]	HRV-A65-A102 [d]		
376	31.25	HRV-A65 [e]	HRV-A65-A102 [d]		
403	31.51	HRV-A65 [e]	HRV-A65-A102 [d]		
693	32.06	HRV-A65 [e]	HRV-A65-A102 [d]		
719	37.43	HRV-A65 [e]	HRV-A65-A102 [d]		
785	24.7	HRV-A65 [e]	HRV-A65-A102 [d]		
789	29.19	HRV-A65 [e]	HRV-A65-A102 [d]		
798	29.09	HRV-A65 [e]	HRV-A65-A102 [d]		
801	30.76	HRV-A65 [e]	HRV-A65-A102 [d]		

Table 3. *Cont.*

Patient ID	CT Value	Genotype	5'UTR	VP4/VP2	other
850	28.86	HRV-A65 [e]	HRV-A65-A102 [d]		
859	30.89	HRV-A65 [e]	HRV-A65-A102 [d]		
879	36.45	HRV-A65 [e]	HRV-A65-A102 [d]		
917	31.61	HRV-A65 [e]	HRV-A65 [f]		
974	30.98	HRV-A65 [e]	HRV-A65-A102 [d]		
998	29.27	HRV-A65 [e]	HRV-A65-A102 [d]		
1001	25.06	HRV-A65 [e]	HRV-A65-A102 [d]		
446	26.82	HRV-A67	HRV-A67		
546	25.33	HRV-A67 [g]		HRV-A67	
559	32.99	HRV-A67	HRV-A67	HRV-A67	
670	29.55	HRV-A67	HRV-A67		
727	38.67	HRV-A67	HRV-A67		
773	33.8	HRV-A67	HRV-A67		
844	29.38	HRV-A67	HRV-A67		
919	27.17	HRV-A67	HRV-A67		
646	34.54	HRV-A71	HRV-A71	HRV-A71	
654	39.24	HRV-A71	HRV-A71		
671	34.58	HRV-A71	HRV-A71	HRV-A71	
436	37.29	HRV-A73	HRV-A73		

Table 3. *Cont.*

Patient ID	CT Value	Genotype	5'UTR	VP4/VP2	other
808	39.86	HRV-A73	HRV-A73		
911	24.7	HRV-A73	HRV-A73		
995	23.92	HRV-A73	HRV-A73		
103	31.16	HRV-A75	HRV-A75	HRV-A75	
144	39.58	HRV-A75	HRV-A75		
250	32.4	HRV-A75	HRV-A75	HRV-A75	
429	37.49	HRV-A78	HRV-A78	HRV-A78	
899	40.33	HRV-A78	HRV-A78	HRV-A78	
962	33.08	HRV-A80	HRV-A80	HRV-A80	
255	28.55	HRV-A81	HRV-A81		
345	34.54	HRV-A81	HRV-A81	HRV-A81	
365	27.72	HRV-A81	HRV-A81		
411	31.06	HRV-A81	HRV-A81		
456	27.21	HRV-A81	HRV-A81		
461	29.7	HRV-A81	HRV-A81		
562	32.15	HRV-A81	HRV-A81	HRV-A81	
655	28.75	HRV-A81		HRV-A81	
708	30.06	HRV-A81		HRV-A81	
740	27.5	HRV-A81	HRV-A81		

Table 3. *Cont.*

Patient ID	CT Value	Genotype	5'UTR	VP4/VP2	other
782	33.22	HRV-A81	HRV-A81		
829	25.31	HRV-A81	HRV-A81		
840	30.68	HRV-A81	HRV-A81		
849	21.94	HRV-A81	HRV-A81	HRV-A81	
861	31.82	HRV-A81	HRV-A81		
864	33.95	HRV-A81	HRV-A81		
900	25.57	HRV-A81	HRV-A81		
921	31.31	HRV-A81	HRV-A81		
925	30.06	HRV-A81	HRV-A81		
988	25.31	HRV-A81	HRV-A81		
116	35.37	HRV-A88	NT	HRV-A88	
598	30.25	HRV-A90	HRV-A90	HRV-A90	
989	38.09	HRV-A90	HRV-A90		
265	22.96	HRV-A96/A61Access nr: KT751298	HRV-A96	HRV-A61	
320	29.71	HRV-A103	HRV-A103		
949	35.89	HRV-A103	HRV-A103	HRV-A103	
984	34.49	HRV-A103	HRV-A103	HRV-A103	
344	30.36	HRV-A106	HRV-A106	HRV-A106	VP1: HRV-A106
713	34.09	HRV-A106	HRV-A106		
CT value, mean ± SD	31.1 ± 4.1				

NT: Not typable; [a] NCBI Genbank entry: KC414927.1; [b] NCBI Genbank entry:FJ445115.1; [c] NCBI Genbank entry: JX129436.1; [d] 5'UTR difficult to discriminate between HRV-A65 and HRV-A102. Finally typed as HRV-A65 based on VP4/VP2; [e] considered as HRV-A65 because it clusters with ID 97T, 123T, 212T, 239T, 304T, 327T and 968T on 5'UTR; [F] NCBI GenBank entry: JF781504.1; [g] Co-infection with EV-99.

Table S4. HRV-B genotyping according to sequenced genome regions.

Patient ID	CT Value	Genotype	5'UTR	VP4/VP2	Other
554	31.72	HRV-B NT	HRV-B NT		
786	34.85	HRV-B NT	HRV-B NT		
39	31.59	HRV-B69	HRV-B69	HRV-B69	
111	31.75	HRV-B69	HRV-B69		
355	23.94	HRV-B69	HRV-B69		
447	34.48	HRV-B69	HRV-B69		
666	28.3	HRV-B69	HRV-B69		
754	26.97	HRV-B69	HRV-B69		
757	30.97	HRV-B69	HRV-B69		
765	37.23	HRV-B69	HRV-B69		
766	33.57	HRV-B69	HRV-B69 [a]		
778	39.95	HRV-B69	HRV-B69		
779	40.49	HRV-B69	HRV-B69		
781	31.05	HRV-B69		HRV-B69	
792	25.61	HRV-B69	HRV-B69		
806	30.51	HRV-B69	HRV-B69		
848	25.8	HRV-B69	HRV-B69		
901	32.53	HRV-B69	HRV-B69		
902	37.15	HRV-B69	HRV-B69	HRV-B69	
930	36.63	HRV-B69	HRV-B69		

Table S4. *Cont*.

Patient ID	CT Value	Genotype	5'UTR	VP4/VP2	Other
940	39.9	HRV-B69	HRV B69		
956	27.31	HRV-B69	HRV-B69	HRV-B69	
1003	29.71	HRV-B69	HRV-B69	HRV-B69	
24	31.19	HRV-B72	NT	HRV-B72	
238	26.97	HRV-B72	NT	HRV-B72	
586	28.5	HRV-B72	HRV-B72 [b]		
629	29.09	HRV-B72	HRV-B72 [b]		
56	34.72	HRV-B79	HRV-B79	HRV-B79	
760	39.05	HRV-B79	HRV-B79 [c]		
108	32.42	HRV-B84	HRV-B84	HRV-B84	
157	38.61	HRV-B84	HRV-B84	HRV-B84	
243	33.86	HRV-B84	HRV-B84		
509	28.41	HRV-B84	HRV-B84		
511	37.72	HRV-B84	HRV-B84		
522	34.57	HRV-B84	HRV-B84		
526	29.53	HRV-B84	HRV-B84		
665	32.3	HRV-B84	HRV-B84	HRV-B84	
17	37.52	HRV-B86	HRV-B86	HRV-B86	
28	38.55	HRV-B86	HRV-B86		
95	36.9	HRV-B86	HRV-B86	HRV-B86	

Table S4. *Cont.*

Patient ID	CT Value	Genotype	5'UTR	VP4/VP2	Other
427	31.58	HRV-B93	HRV-B93	HRV-B93	
736	32	HRV-B_Pat09	HRV-B72 Access nr: KR997889 87% with JN798562.2	HRV-B27 Access nr: KR997886 85% with FJ445186.1	VP2/VP3: HRV-B84 Access nr: KR997887 78% with JQ837723.1
CT value, mean ± SD	32.8 ± 4.4				

NT: Not typable; [a]NCBI GenBank entry: JQ245970.1; [b] NCBI GenBank entry: JN798562.2; [c] NCBI GenBank entry: EU096066.1.

Table S5. HRV-C genotyping according to sequenced genome regions.

Patient ID	CT Value	Genotype	5'UTR	VP4/VP2	other
76	33.84	HRV-C NT			
328	32.64	HRV-C NT			
696	34.87	HRV-C NT			
398	25.53	HRV-C1	HRV-C1	HRV-C1	
261	27.31	HRV-C2	HRV-C2	HRV-C2	
383	23.03	HRV-C2	HRV-C2	HRV-C2	
392	28.69	HRV-C2	HRV-C2	HRV-C2	
413	35.31	HRV-C2	HRV-C2	HRV-C2	
424	35.04	HRV-C2	HRV-C2	HRV-C2	
499	31.94	HRV-C2	HRV-C2	HRV-C2	
569	NA	HRV-C2	HRV-C2	HRV-C2	
579	28.72	HRV-C2	HRV-C2		
628	30.07	HRV-C2	HRV-C2	HRV-C2	

Table S5. *Cont.*

Patient ID	CT Value	Genotype	5'UTR	VP4/VP2	other
635	26.59	HRV-C2	HRV-C2	HRV-C2	
5	32.79	HRV-C6	HRV-C6	HRV-C6	
55	25.99	HRV-C6	HRV-C6	HRV-C6	
814	39.81	HRV-C6	HRV-C6	HRV-C6	
484	38.41	HRV-C7	HRV-C7		
507	31.16	HRV-C7	HRV-C7	HRV-C7	
739	27.69	HRV-C7	HRV-C7	HRV-C7	
838	23.04	HRV-C7	HRV-C7	HRV-C7	
882	32.68	HRV-C13	HRV-C13 [a] Access nr: KR997890	HRV-C13	
886	25.97	HRV-C13	HRV-C13 [a]	HRV-C13	
950	33.67	HRV-C13	HRV-C13 [a]	HRV-C13	
1000	27.27	HRV-C13	HRV-C13 [a]	HRV-C13	
688	34.84	HRV-C15	HRV-C15		
122	30.23	HRV-C16	HRV-C16 [a]	HRV-C16	VP1: HRV-C16
639	32.49	HRV-C16	HRV-C16 [a] Access nr: KR997882	HRV-C16	VP1: HRV-C16
330	25.58	HRV-C17	HRV-C17		
334	37.3	HRV-C17	HRV-C17		
610	33.6	HRV-C17	HRV-C17	HRV-C17	VP1: HRV-C17
701	28.84	HRV-C18	HRV-C18 [a] Access nr: KR997891	HRV-C18	
769	25.6	HRV-C18	HRV-C18[a]	HRV-C18	

Table S5. *Cont.*

Patient ID	CT Value	Genotype	5'UTR	VP4/VP2	other
851	35.58	HRV-C18	HRV-C18[a]	HRV-C18	
938	36.37	HRV-C18	HRV-C18[a]	HRV-C18	
982	27.8	HRV-C18	HRV-C18[a]	HRV-C18	
749	31.84	HRV-C18	HRV-C18[a, b]		
804	28.6	HRV-C18	HRV-C18[a, b]		
836	26.21	HRV-C18	HRV-C18[a, b]		
638	29.2	HRV-C18	HRV-C18[c]	HRV-C18	
539	32.2	HRV-C19	HRV-C19[a] Access nr: KR997892	HRV-C19	
530	37.41	HRV-C19	HRV-C19[a, d]		
568	35.72	HRV-C19	HRV-C19[a, d]		
237	24.06	HRV-C22	NT	HRV-C22	
248	28.09	HRV-C22	NT	HRV-C22	
368	29.55	HRV-C22	NT	HRV-C22	
489	33.46	HRV-C22	NT	HRV-C22	
622	21.68	HRV-C22	NT	HRV-C22	
653	33.51	HRV-C22	NT	HRV-C22	
288	20.83	HRV-C23	HRV-C23 [a] Access nr: KR997881	HRV-C23	VP3: HRV-C23 [e] Access nr: KR997881
615	26.71	HRV-C23	HRV-C23 [a, f]		
119	35.97	HRV-C27	HRV-C27 [a] Access nr: KR997893	HRV-C27	
387	30.35	HRV-C27	HRV-C27[a]	HRV-C27	

Table S5. *Cont.*

Patient ID	CT Value	Genotype	5'UTR	VP4/VP2	other
558	21.84	HRV-C27	HRV-C27[a]	HRV-C27	
342	36.4	HRV-C27	HRV-C27 [a, g]		
566	39.54	HRV-C33	HRV-C33 [a] Access nr: KR997894	HRV-C33	
606	38.74	HRV-C33	HRV-C33[a, h]		
390	21.23	HRV-C40		HRV-C40	
466	34.47	HRV-C40	HRV-C40	HRV-C40	
839	31.92	HRV-C40	HRV-C40	HRV-C40	
841	24.16	HRV-C40	HRV-C40		
322	32.91	HRV-C42	HRV-C42	HRV-C42	
401	26.91	HRV-C42	HRV-C42	HRV-C42	
987	31.56	HRV-C46	HRV-C46 [a] Access nr: KR997880	HRV-C46	
543	39.66	HRV-C50	HRV-C50	HRV-C50	
464	28.72	HRV-C51		HRV-C51	
529	36.59	HRV-C55	HRV-C35 92% with JF436925.1	HRV-C35 80% with EU081790.1	5'UTR/2A: HRV-C35 Access nr: KR997885 81% with JF436925.1
583	27.93	HRV-C55	HRV-C35 92% with JF436925.1	HRV-C35 80% with EU081790.1	
584	38.83	HRV-C55	HRV-C35 92% with JF436925.1		
626	39.67	HRV-C55	HRV-C35 92% with JF436925.1	HRV-C35 80% with EU081790.1	
668	38.61	HRV-C55	HRV-C35 92% with JF436925.1		
624	38.4	HRV-C_Pat22 [i]	HRV-C54 Access nr: KR997884 81% with KP282614.1	HRV-C48 Access nr: KR997884 85% with JF519763	VP2/VP3: HRV-C28 Access nr: KR997888 74% with JN798569.1

Table S5. *Cont.*

Patient ID	CT Value	Genotype	5'UTR	VP4/VP2	other
617	32.19	HRV-C_Pat22 [i]	HRV-C54 81% with KP282614.1	HRV-C48 85% with JF519763	
644	38.57	HRV-C_Pat22 [i]	HRV-C54 81% with KP282614.1	HRV-C48 85% with JF519763	
710	31.6	HRV-C_Pat22 [i]	HRV-C54 81% with KP282614.1		
813	39.65	HRV-C_Pat21 [j]	HRV-C36 91% with KF499421.1	HRV-C36 85% with KF688657.1	5'UTR/VP3: HRV-C36 Access nr: KR997883 84% with JN541267.1
CT value, mean ± SD	31.9 ± 5.2				

NA: Not available; NT: Not typable;[a] New 5'UTR sequences provided for these serotypes; [b] Considered as HRV-C18 because it clusters with ID 701T, 769T, 851T, 938T and 982T on 5'UTR; [c] NCBI GenBank entry: KF499468.1; [d] Considered as HRV-C19 because it clusters with ID 539T on 5'UTR; [e] New VP3 sequence provided for HRV-C23; [f] Considered as HRV-C23 because it clusters with ID 288T on 5'UTR; [g] Considered as HRV-C27 because it clusters with ID 119T, 387T and 558T on 5'UTR; [h] Considered as HRV-C33 because it clusters with ID 566T on 5'UTR; [i] NCBI GenBank entry: FJ615745; [j] NCBI GenBank entry: FJ615737.

Table S6. HEV-A genotyping according to sequenced genome regions.

Patient ID	CT Value	Genotype	5'UTR	VP4/VP2	VP1
893	33.49	CV-A2	NT	CV-A2	
416	36.4	CV-A4	NT	CV-A4	
672	32.16	CV-A4	NT	CV-A4	
842	28.1	CV-A4	NT	CV-A4	
399	37.56	CV-A6	NT	CV-A6	CV-A6
3	39.12	CV-A8	NT	CV-A8	
431	35.92	CV-A10	NT	CV-A10	CV-A10
48	40.68	CV-A10/A8	CV-A10	CV-A8	
477	36.01	CV-A10/A8	CV-A10	CV-A8	

Table S6. *Cont.*

Patient ID	CT Value	Genotype	5'UTR	VP4/VP2	VP1
922	30.08	HEV-A71	CV-A8	HEV-A71	HEV-A71
996	37.13	HEV-A71	CV-A8	HEV-A71	HEV-A71
CT value, mean ± SD	35.2 ± 3.8				
NT: Not typable.					

Table S7. HEV-B genotyping according to sequenced genome regions.

Patient ID	CT Value	Genotype	5'UTR	VP4/VP2	VP1
857	34.72	CV-A9	NT	CV-A9	
909	37.53	CV-B1	NT	NT	CV-B1 [a]
862	32.23	CV-B1 [b]	NT	NT	
952	40.62	CV-B1 [b]	NT	NT	
955	35.49	CV-B1 [b]	NT	NT	
822	35.67	CV-B2	NT	CV-B2	
888	32.37	CV-B2	HEV-B106	CV-B2	CV-B2
894	36.75	CV-B2	HEV-B106	CV-B2	CV-B2
895	36.96	CV-B2	HEV-B106	CV-B2	CV-B2
941	40.8	CV-B2	NT	CV-B2	
777	37.11	CV-B5	NT	NT	CV-B5
860	31.94	CV-B5	NT	NT	CV-B5
871	35.62	CV-B5	NT	NT	CV-B5

Table S7. *Cont.*

Patient ID	CT Value	Genotype	5'UTR	VP4/VP2	VP1
904	40.8	CV-B5	NT	NT	CV-B5
961	30.73	CV-B5	NT	NT	CV-B5
977	37.28	CV-B5	NT	NT	CV-B5
1005	30.68	CV-B5	NT	NT	CV-B5
1007	29.58	CV-B5	NT	NT	CV-B5
887	35.97	CV-B5 [c]	NT	NT	
932	32.49	CV-B5 [c]	NT	NT	
313	40.18	E-6	NT	NT	E-6
162	40.9	E-7	NT	NT	E-7
951	26.56	E-9	NT	NT	E-9
407	37.92	E-9 [d]	NT	NT	
807	34.33	E-9 [d]	NT	NT	
825	27.37	E-9 [d]	NT	NT	
866	32.11	E-9 [d]	NT	NT	
994	25.93	E-9 [d]	NT	NT	
863	37.75	E-11	NT	NT	E-11
CT value, mean ± SD	34.8 ± 4.3				

NT: Not Typable; [a] NCBI GenBank entry: KJ472841.1; [b] Considered as CV-B1 because it clusters with 909T on 5'UTR and VP4/VP2; [c] Considered as CV-B5 because it clusters with 777T, 860T, 871T, 904T, 932T, 961T, 977T, 1005T and 1007T on 5'UTR and VP4/VP2; [d] Considered as E-9 because it clusters with 951T on 5 'UTR and VP4/VP2.

Table S8. HEV-C genotyping according to sequenced genome regions.

Patient ID	CT Value	Genotype	5'UTR	VP4/VP2	Other
590	38.05	CV-A13	NT	NT	VP1: CV-A13 [a]
648	38.57	CV-A13	CV-A13	NT	VP1: CV-A13
207	39.03	CV-A24	NT [b]	CV-A24	
546	25.33	HEV-C99 [c]		HEV-C99	
593	33.36	PV Sabin1	PV1A Access nr: KR997879	PV1A Access nr: KR997879	PV1A [d,e] VP3/VP1: Access nr: KR9978952C: Access nr: KR9978963A/3C: Access nr: KR997897
948	39.32	NT	NT	NT	
CT value, mean ± SD	35.6 ± 5.5				

NT: Not typable; [a] NCBI GenBank entry: JF260920.1; [b] The first 787 nucleotides had 97% similarity with CV-A24 (GenBank entry: EU221292.1); [c] Co-infection with HRV-A67; [d] NCBI GenBank entry: AB467606.1; [e] NCBI GenBank entry: KJ170532.1.

Table S9. HEV-D genotyping according to sequenced genome regions.

Patient ID	CT Value	Genotype	5'UTR	VP4/VP2
202	32.86	HEV-D68	HEV-D68	HEV-D68
487	32.11	HEV-D68	HEV-D68	HEV-D68
525	35.96	HEV-D68	HEV-D68	HEV-D68
CT value, mean ± SD	33.6 ± 2.0			

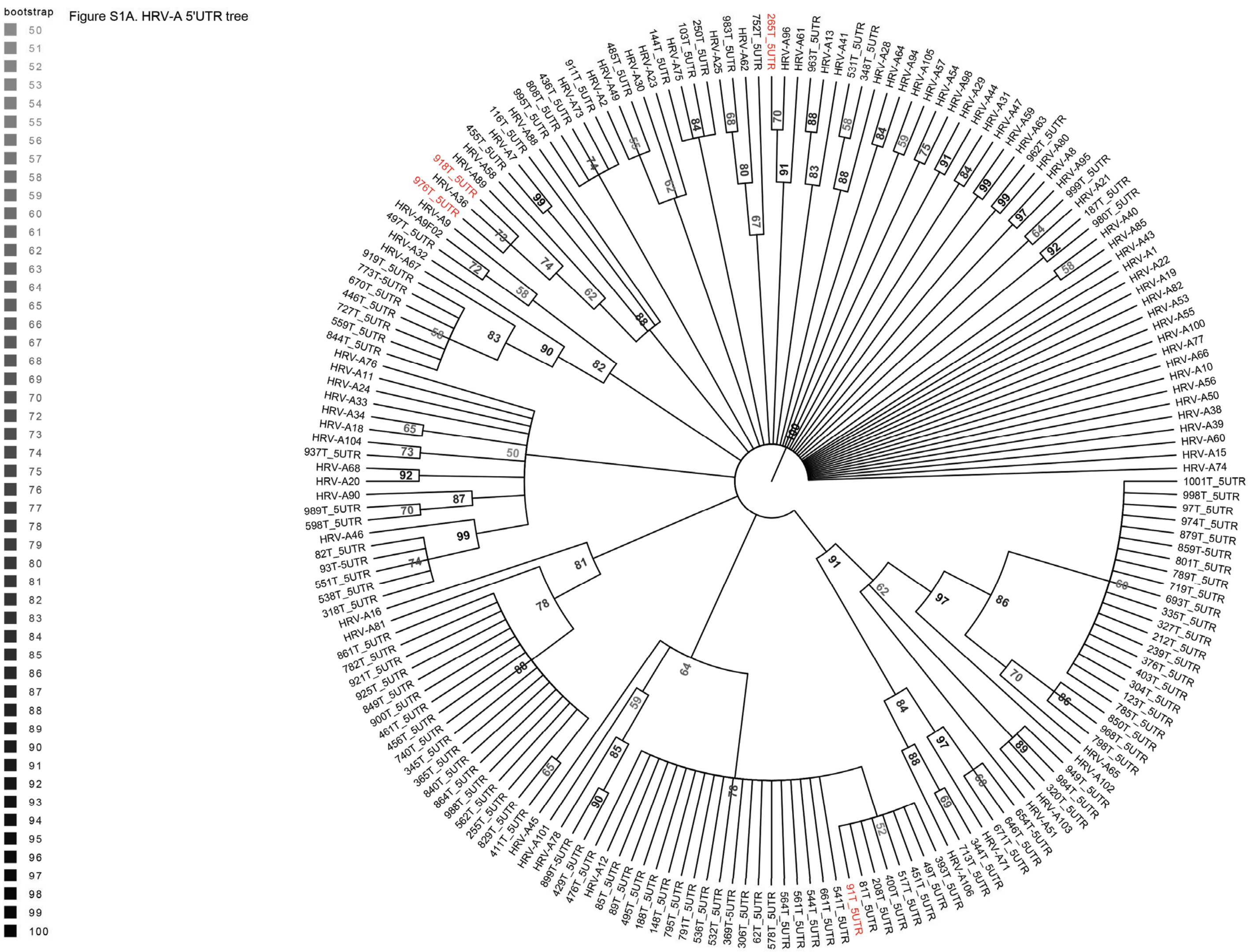

(A)

Figure S1. *Cont*.

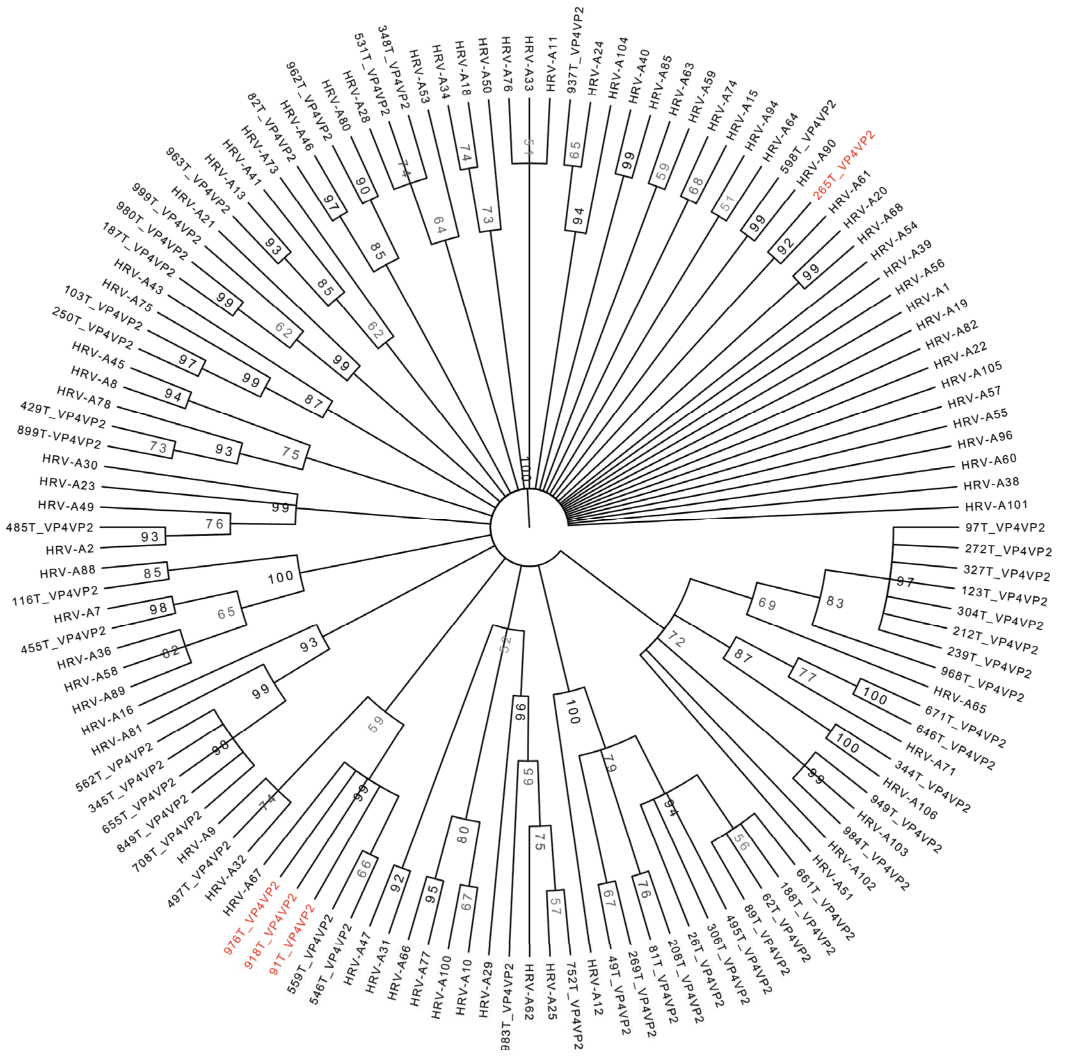

(B)

Figure S1. (**A**) HRV-A 5'UTR tree (nt 240 to 449 relative to HRV-A2_X02316); (**B**) HRV-A VP4/VP2 tree (nt 611 to 1030 relative to HRV-A2_X02316).

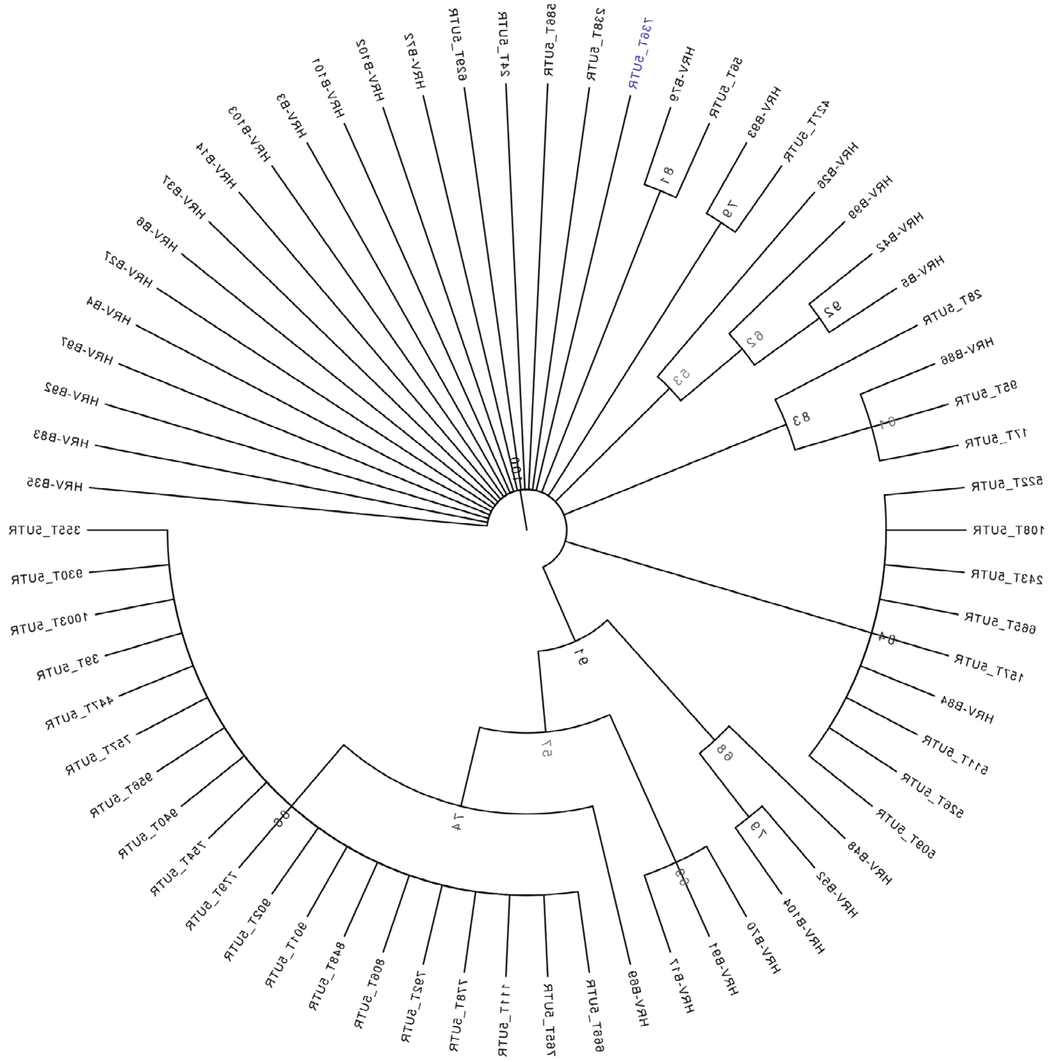

(**A**)

Figure S2. *Cont*.

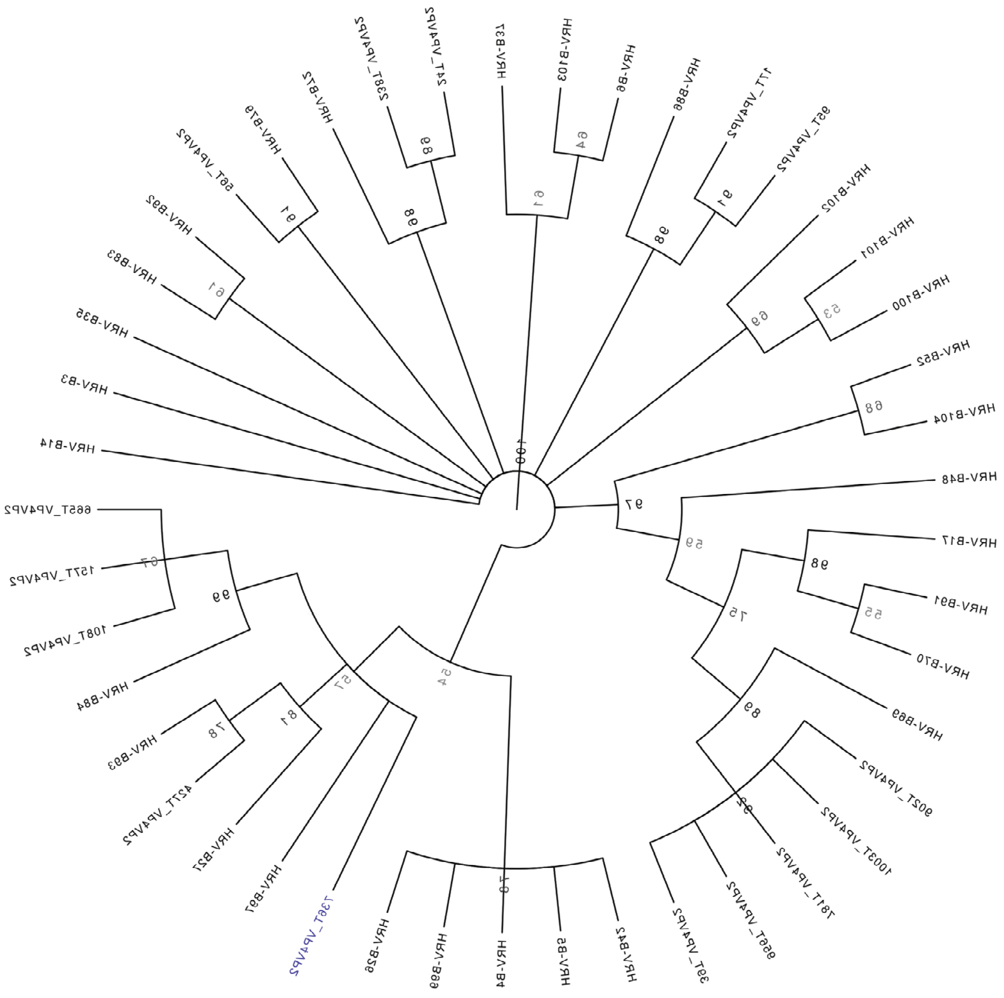

(**B**)

Figure S2. (**A**) HRV-B 5′UTR tree (nt 177 to 449 relative to HRV-A2_X02316); (**B**) HRV-B VP4/VP2 tree (nt 611 to 1018 relative to HRV-A2_X02316).

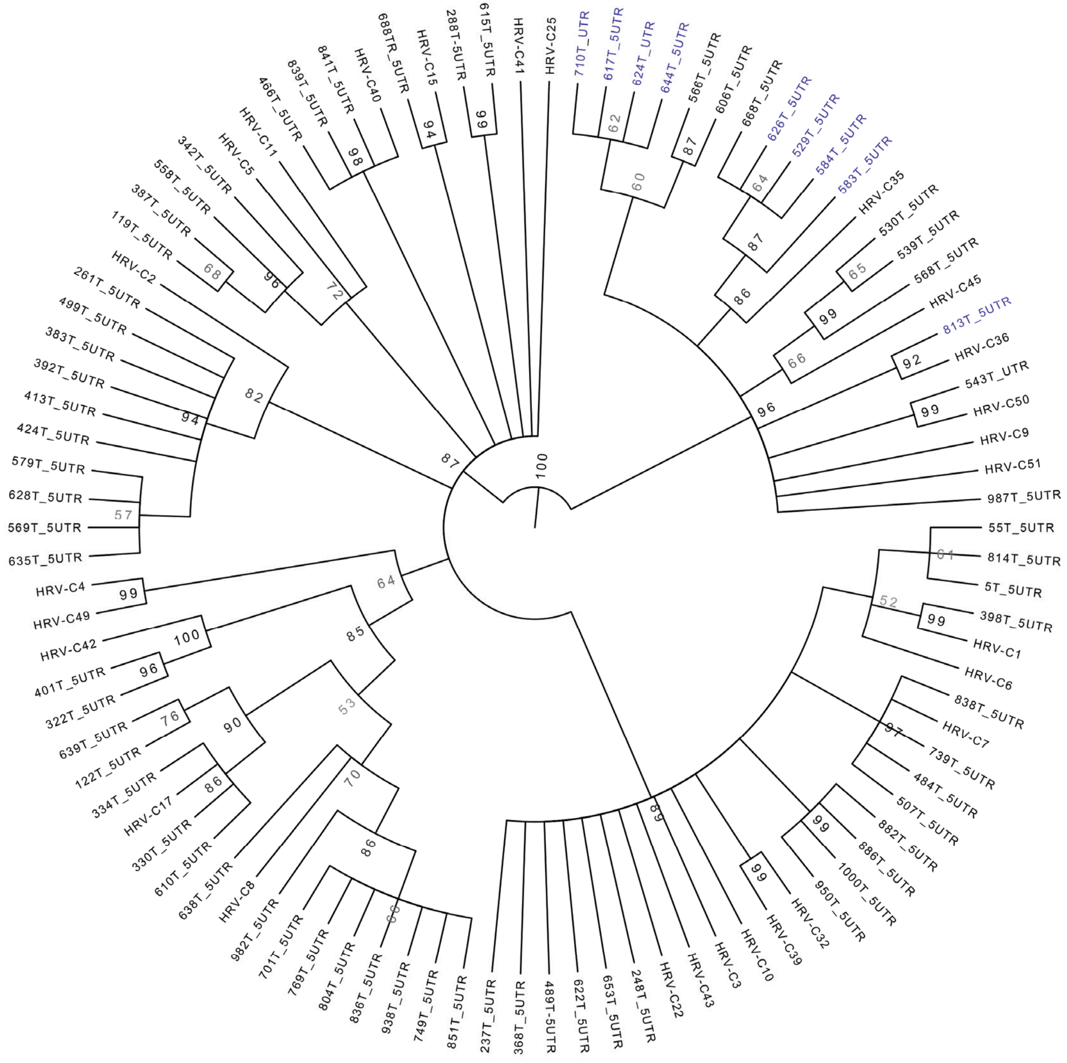

(A)

Figure S3. *Cont*.

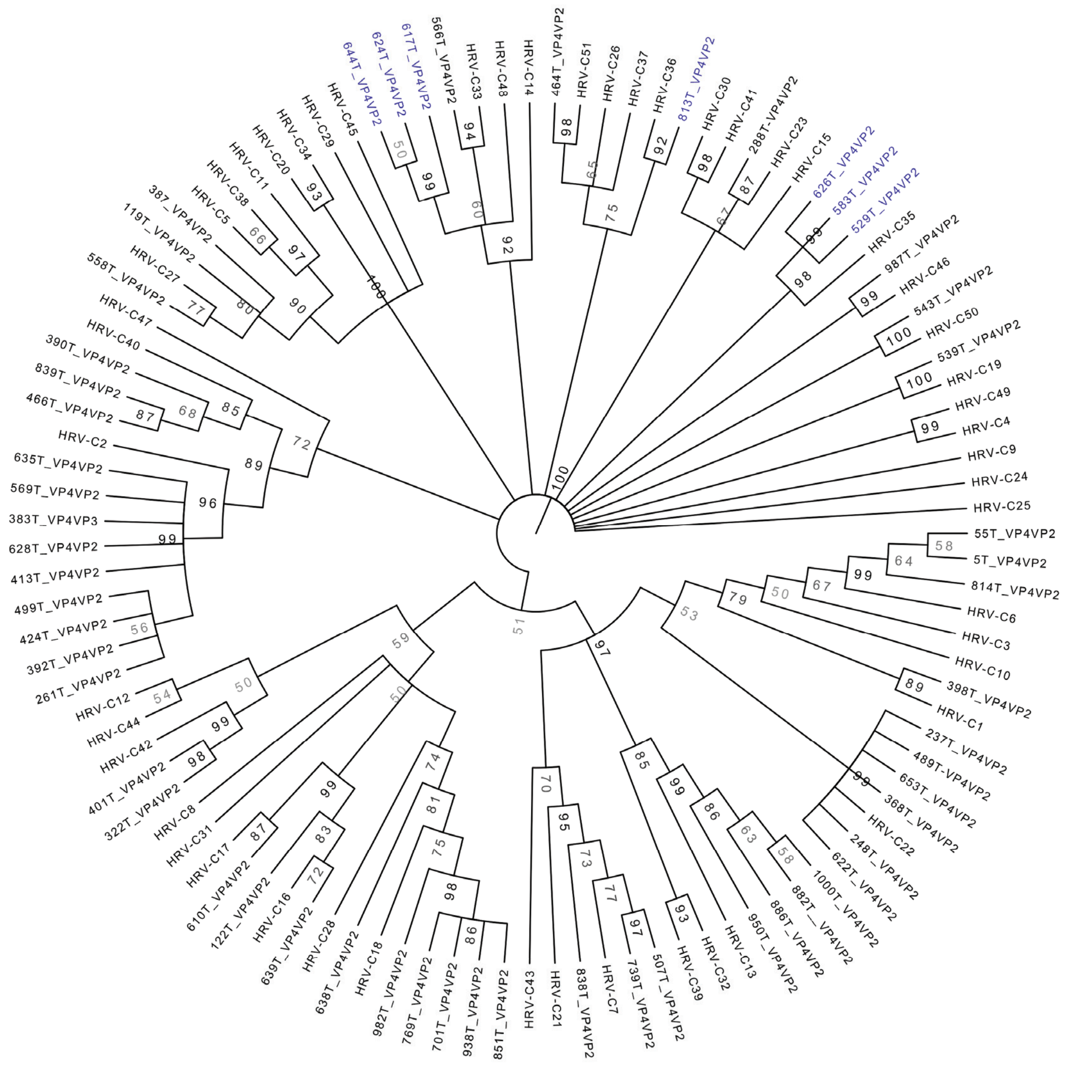

(B)

Figure S3. (**A**) HRV-C 5′UTR tree (nt 240 to 449 relative to HRV-A2_X02316); (**B**) HRV-C VP4/VP2 tree (nt 611 to 1075 relative to HRV-A2_X02316).

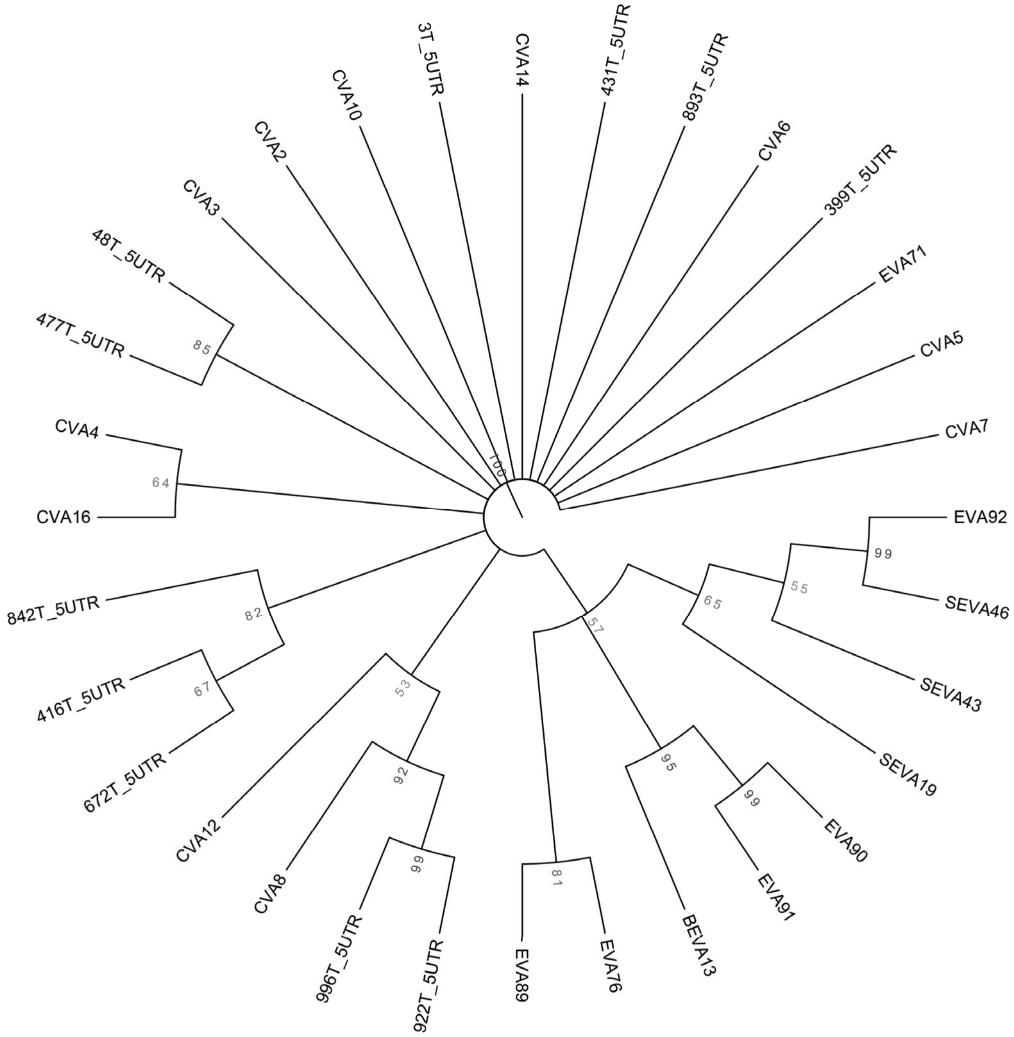

(A)

Figure S4. *Cont*.

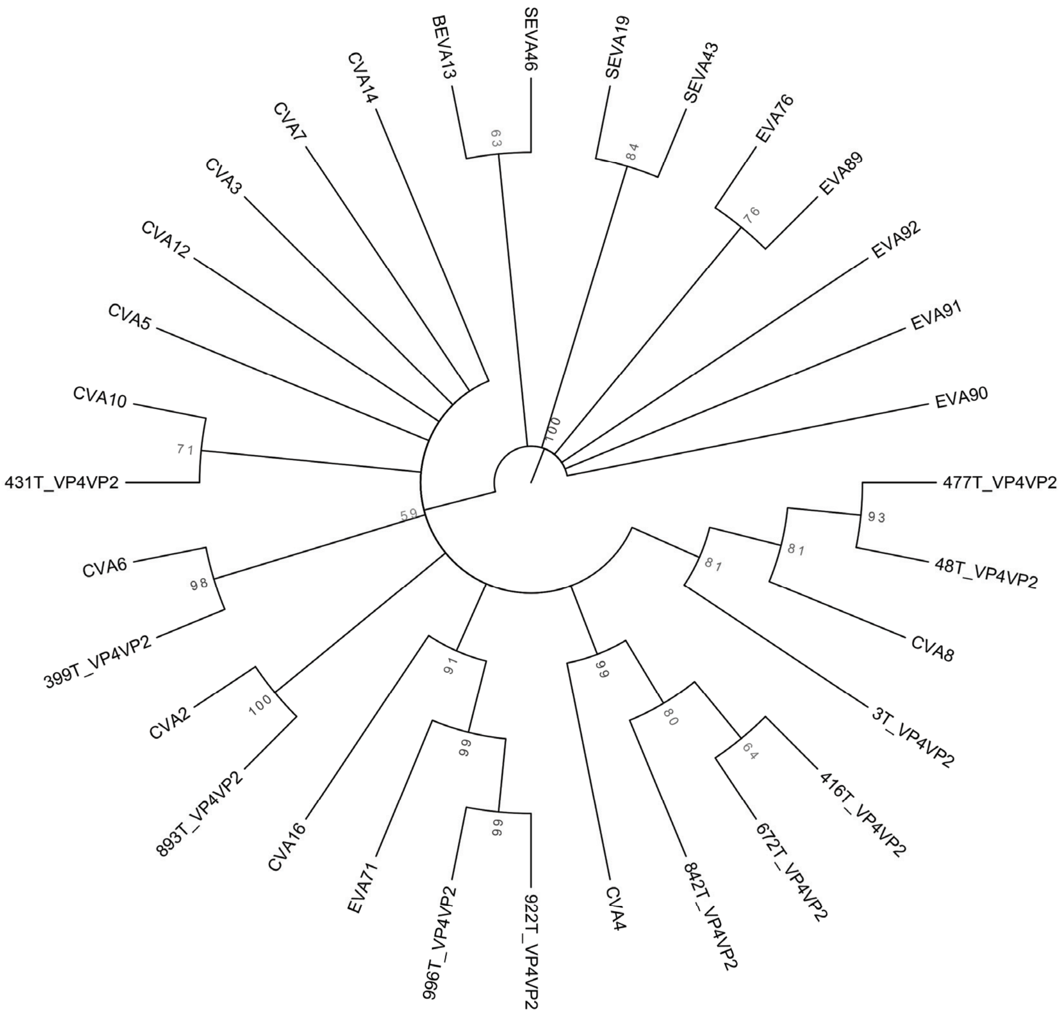

(B)

Figure S4. *Cont*.

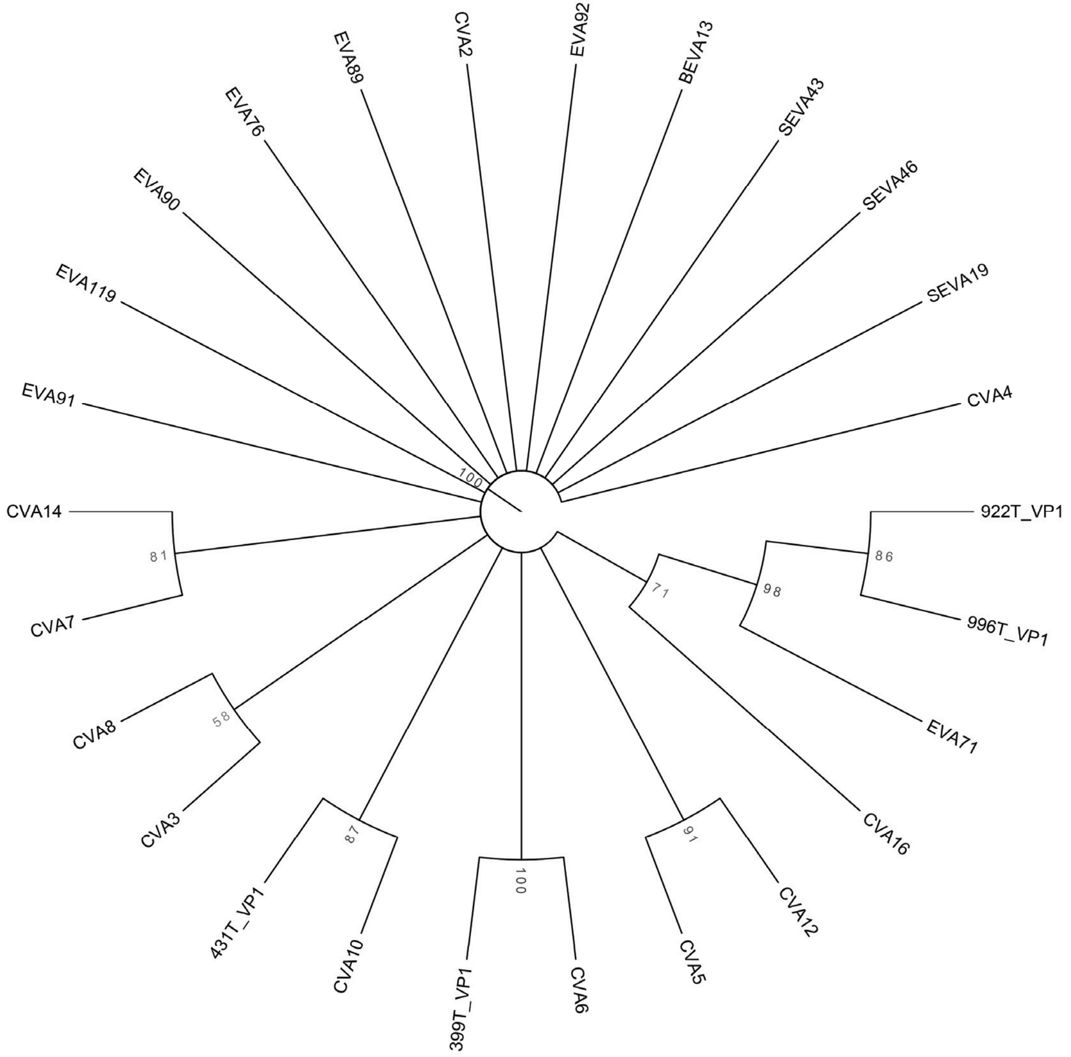

(C)

Figure S4. *Cont*.

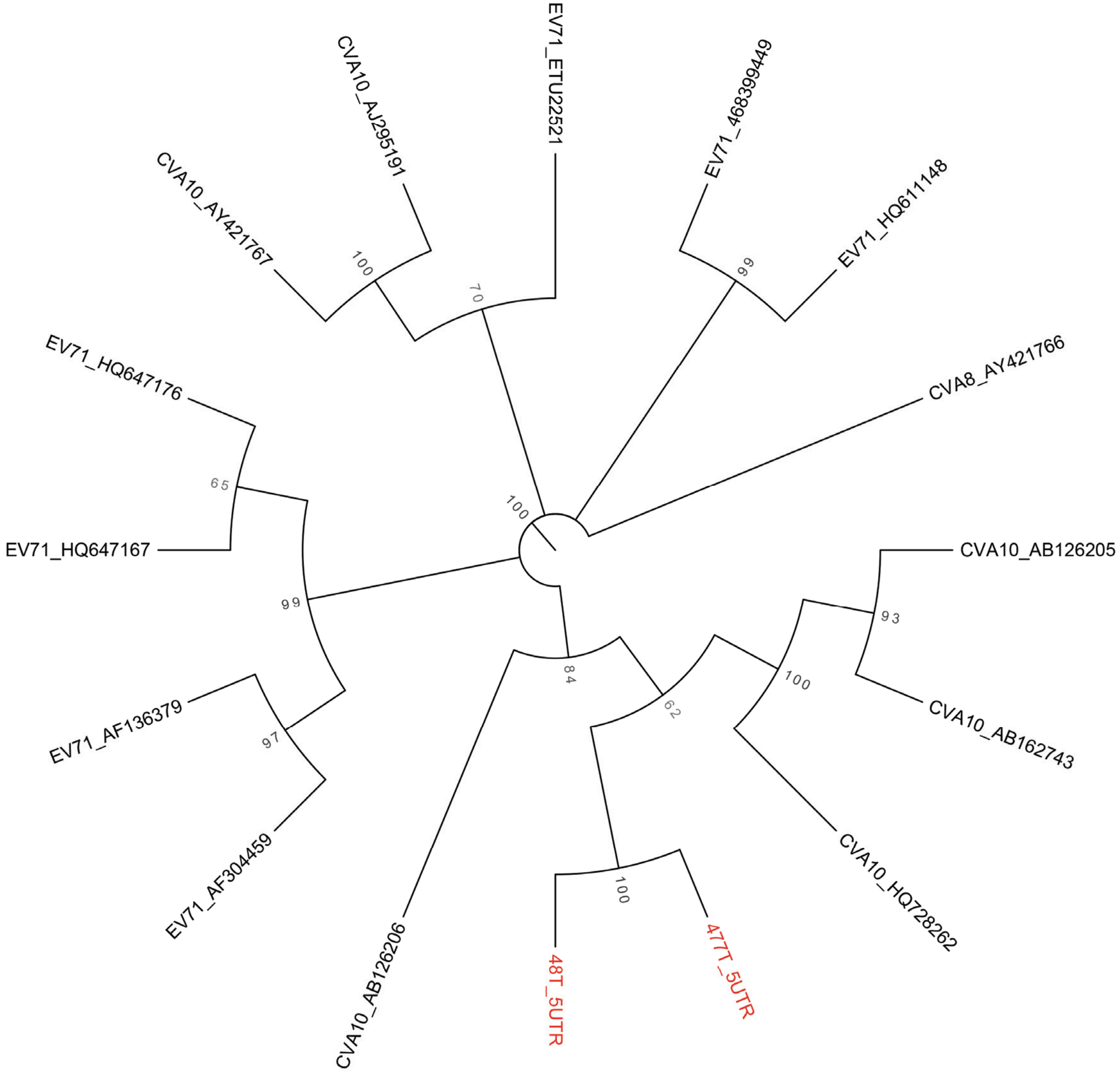

(D)

Figure S4. *Cont*.

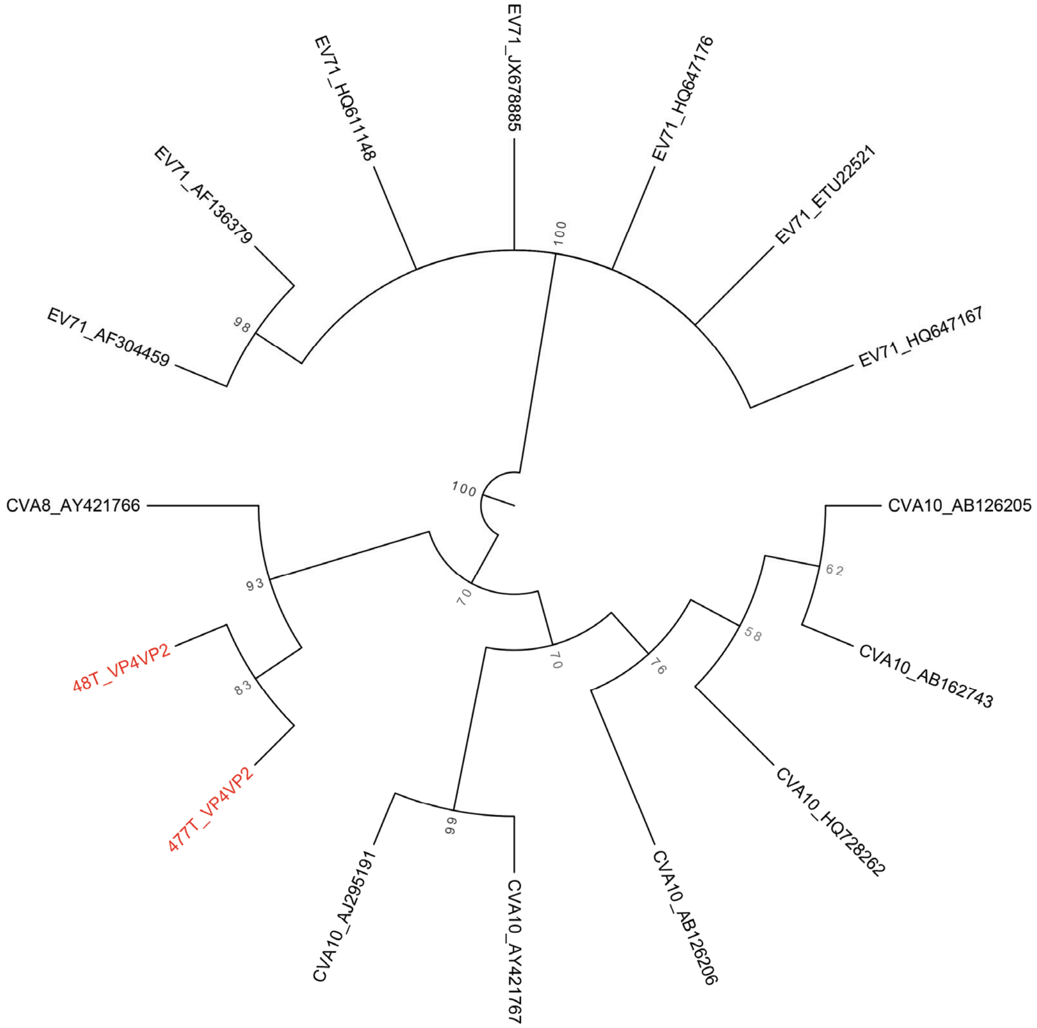

(**E**)

Figure S4. (**A**) HEV-A 5'UTR tree (nt 177 to 370 relative to HRV-A2_X02316); (**B**) HEV-A VP4/VP2 tree (nt 611 to 1030 relative to HRV-A2_X02316); (**C**) HEV-A VP1 tree (nt 2429 to 2709 relative to HRV-A2_X02316); (**D**) HEV-A 5'UTR recombinant tree (nt 169 to 611 relative to HRV-A2_X02316); (**E**) HEV-A VP4/VP2 recombinant tree (nt 611 to 1038 relative to HRV-A2_X02316).

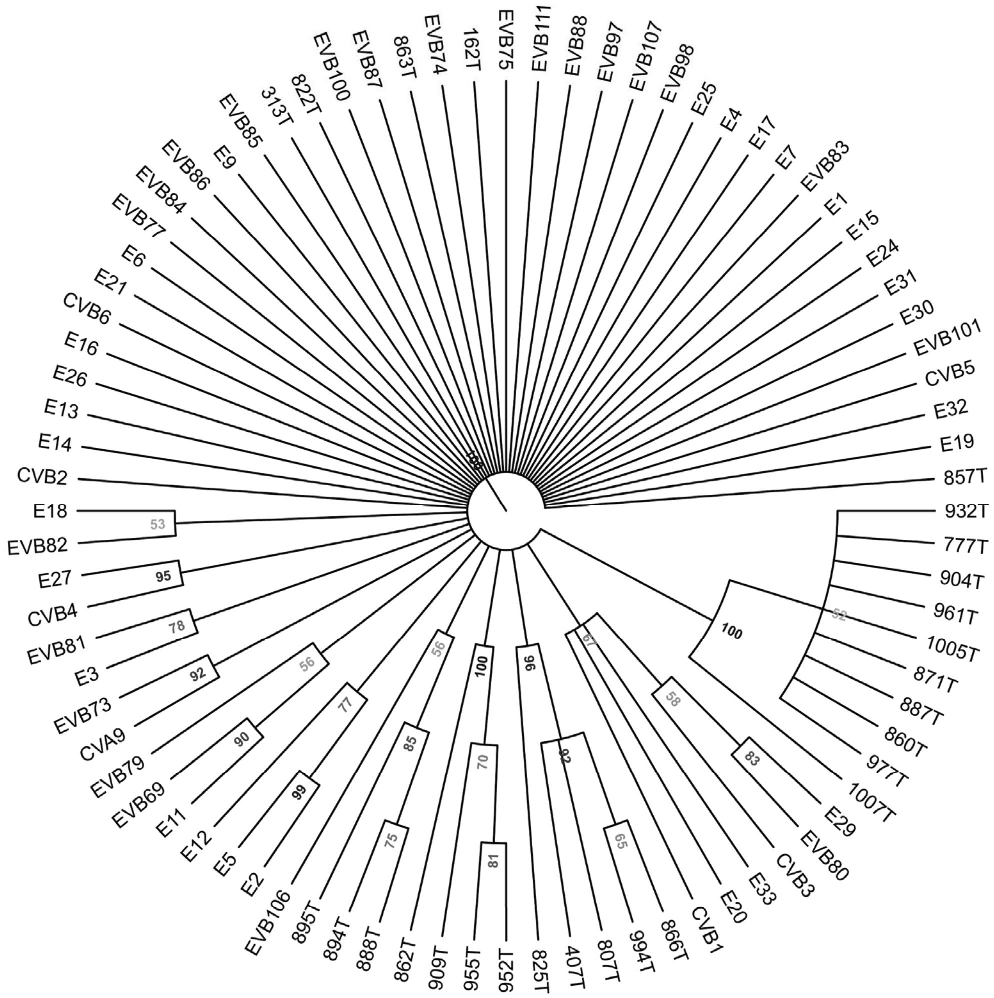

(**A**)

Figure S5. *Cont*.

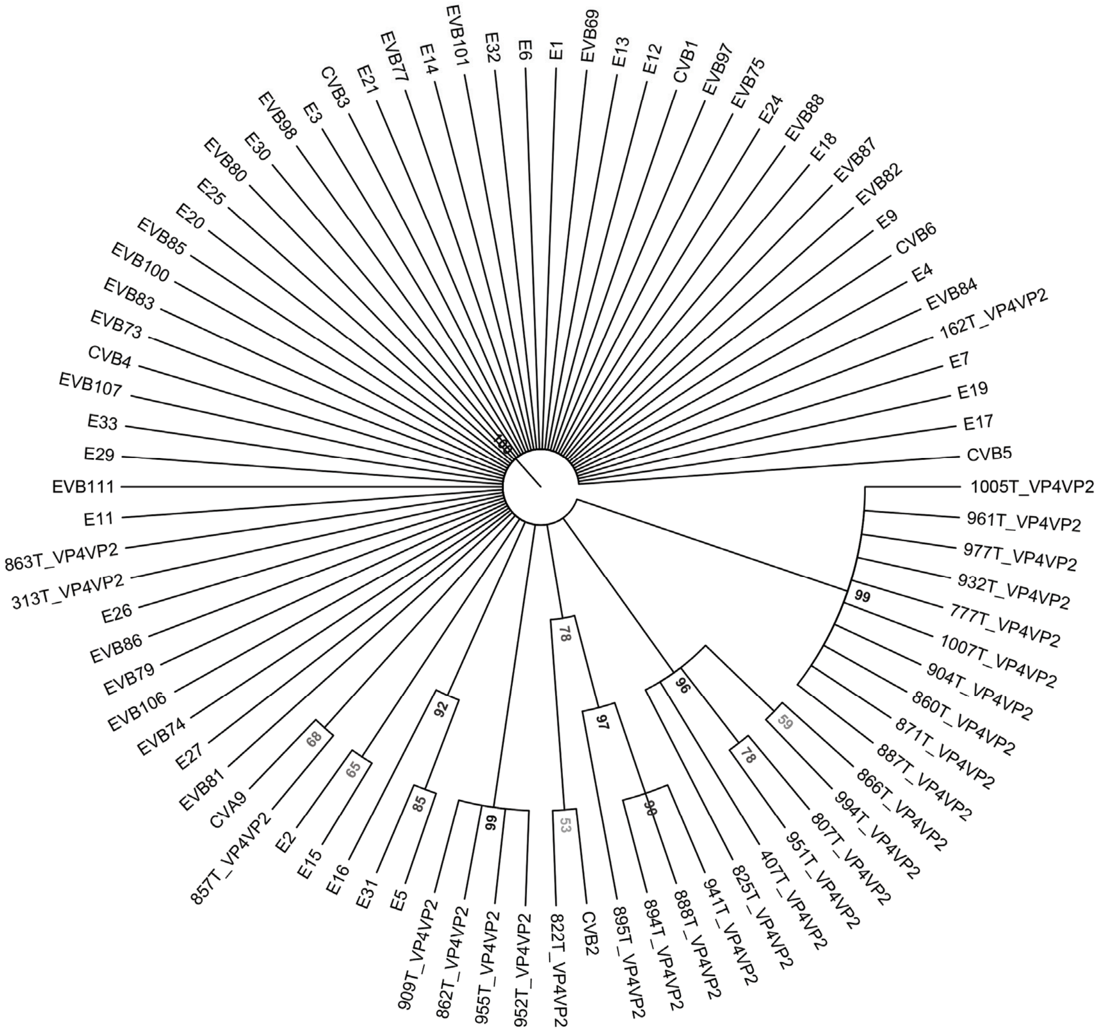

(**B**)

Figure S5. *Cont*.

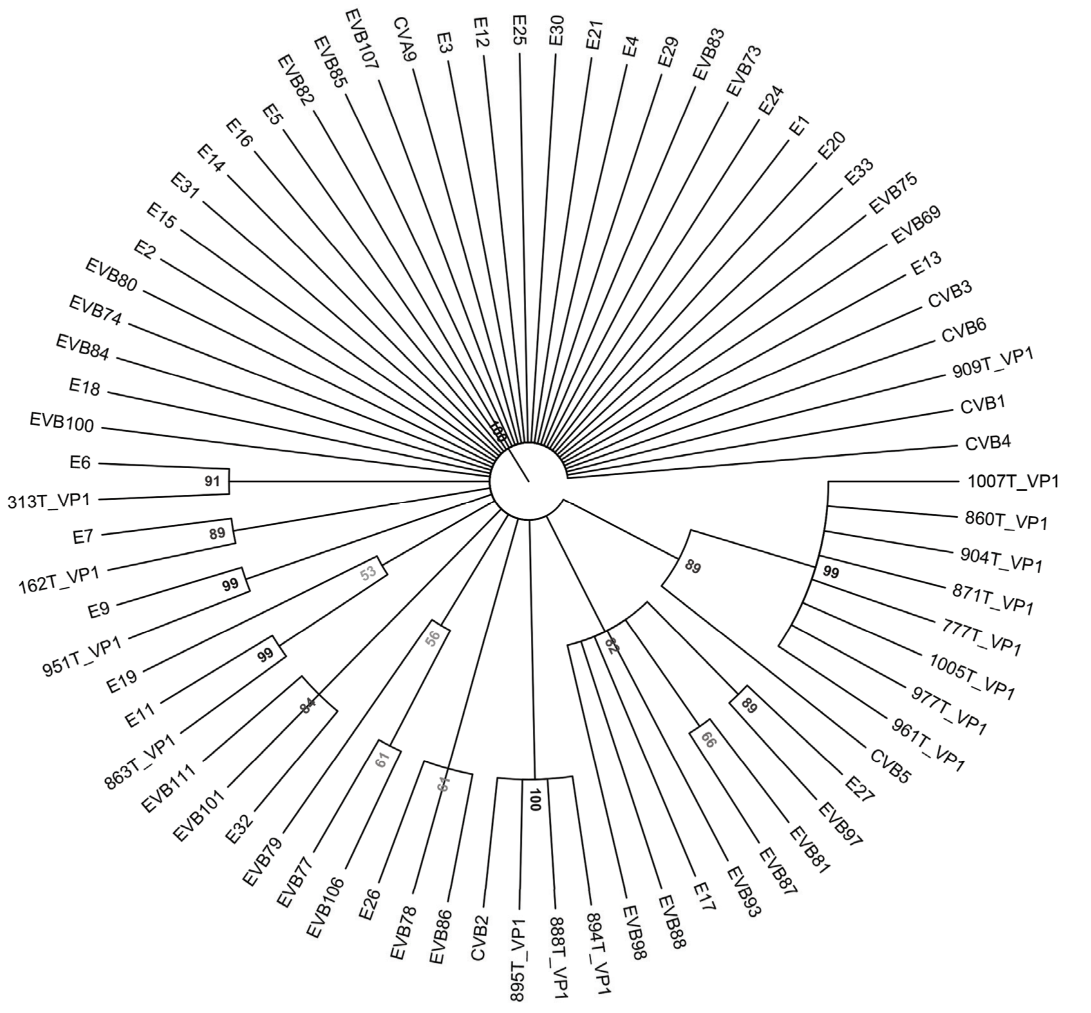

(**C**)

Figure S5. (**A**) HEV-B 5'UTR tree (nt 193 to 611 relative to HRV-A2_X02316); (**B**) HEV-B VP4/VP2 tree (nt 661 to 993 relative to HRV-A2_X02316); (**C**) HEV-B VP1 tree (nt 2429 to 2705 relative to HRV-A2_X02316).

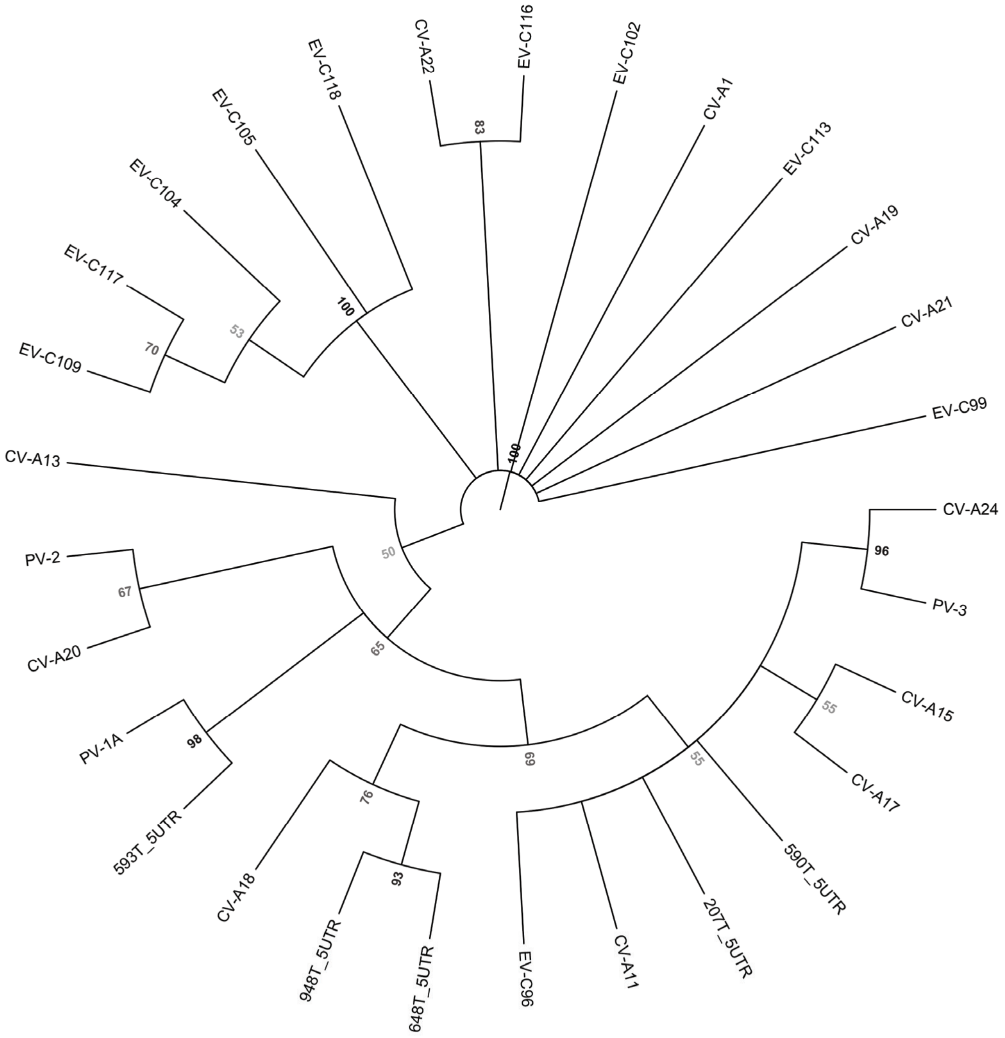

(**A**)

Figure S6. *Cont*.

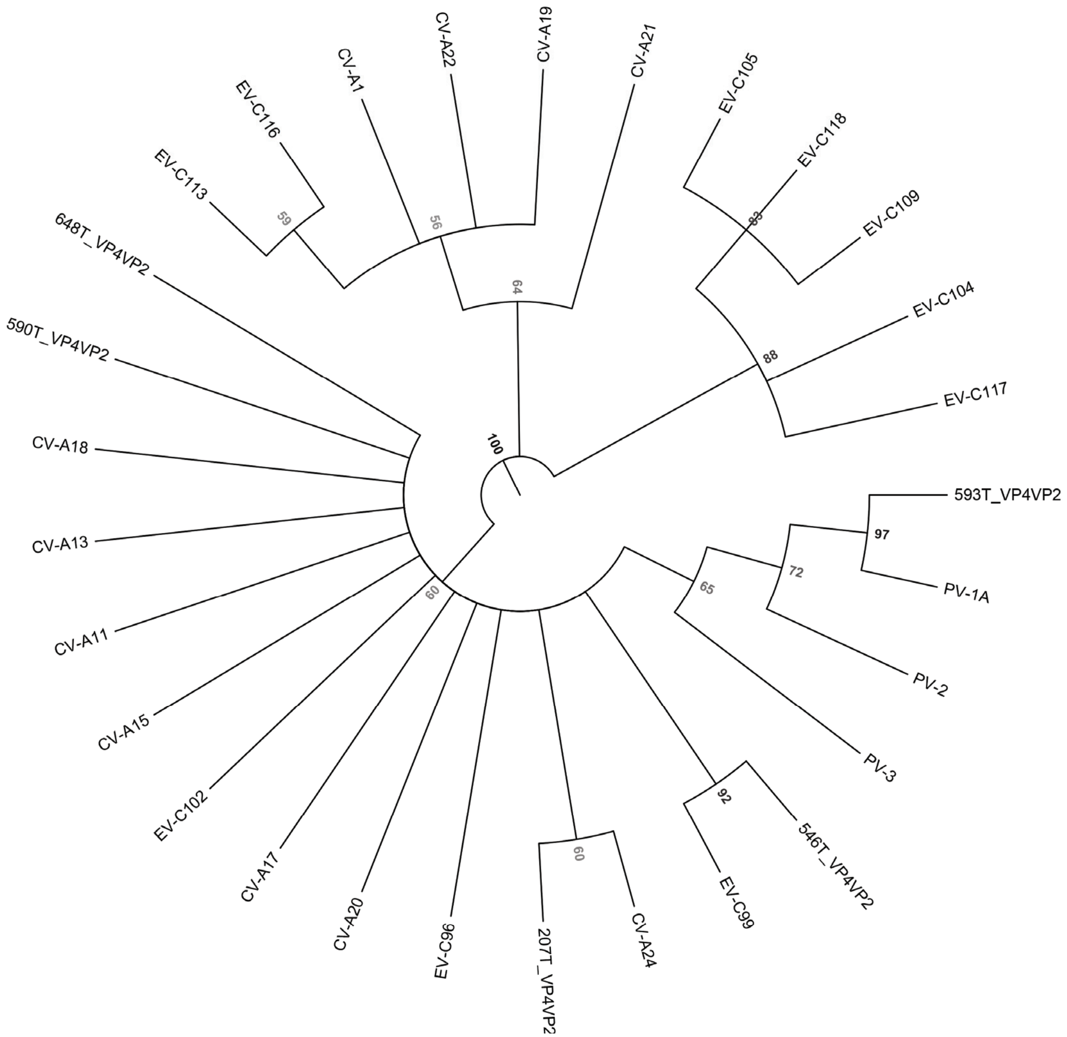

(**B**)

Figure S6. *Cont*.

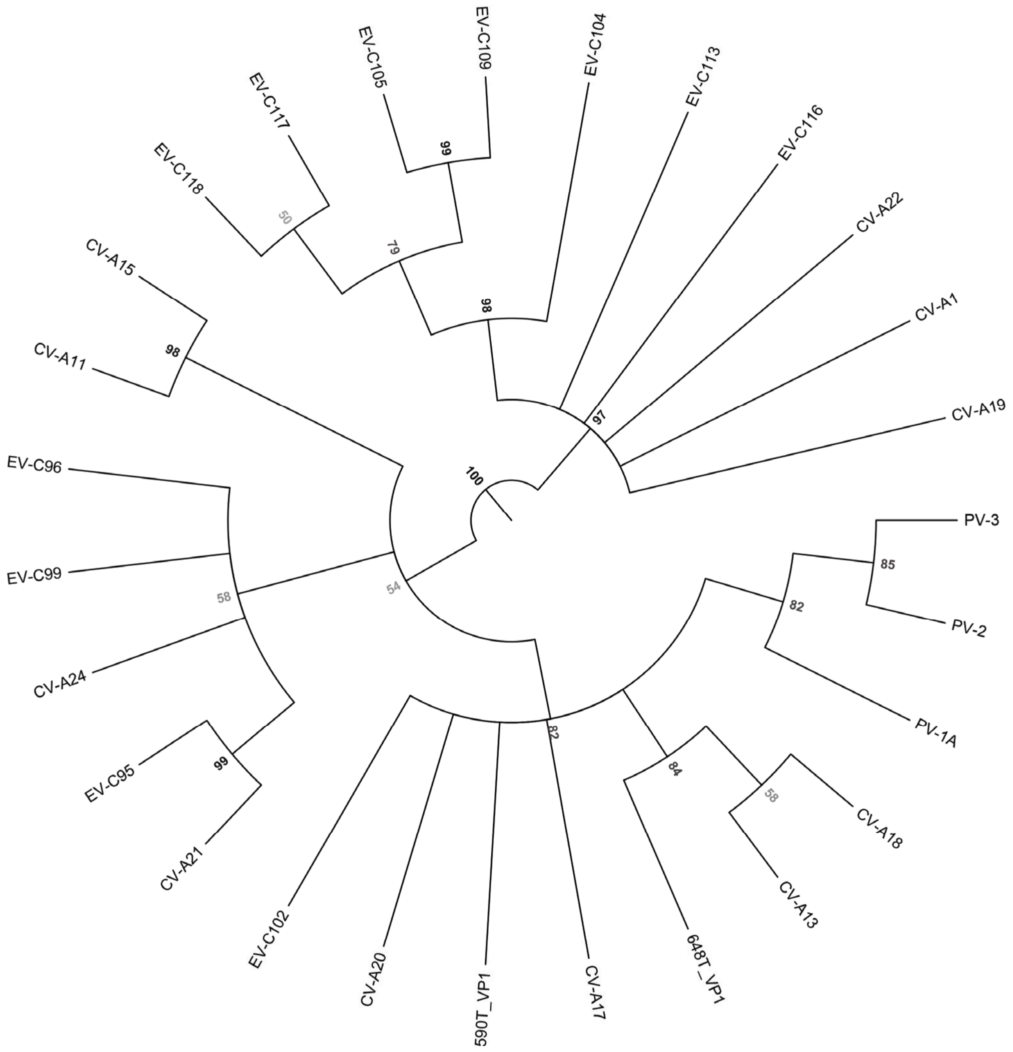

(**C**)

Figure S6. (**A**) HEV-C 5'UTR tree (nt 281 to 557 relative to HRV-A2_X02316); (**B**) HEV-C VP4/VP2 tree (nt 611 to 934 relative to HRV-A2_X02316); (**C**) HEV-C VP1 tree (nt 2450 to 2743 relative to HRV-A2_X02316).

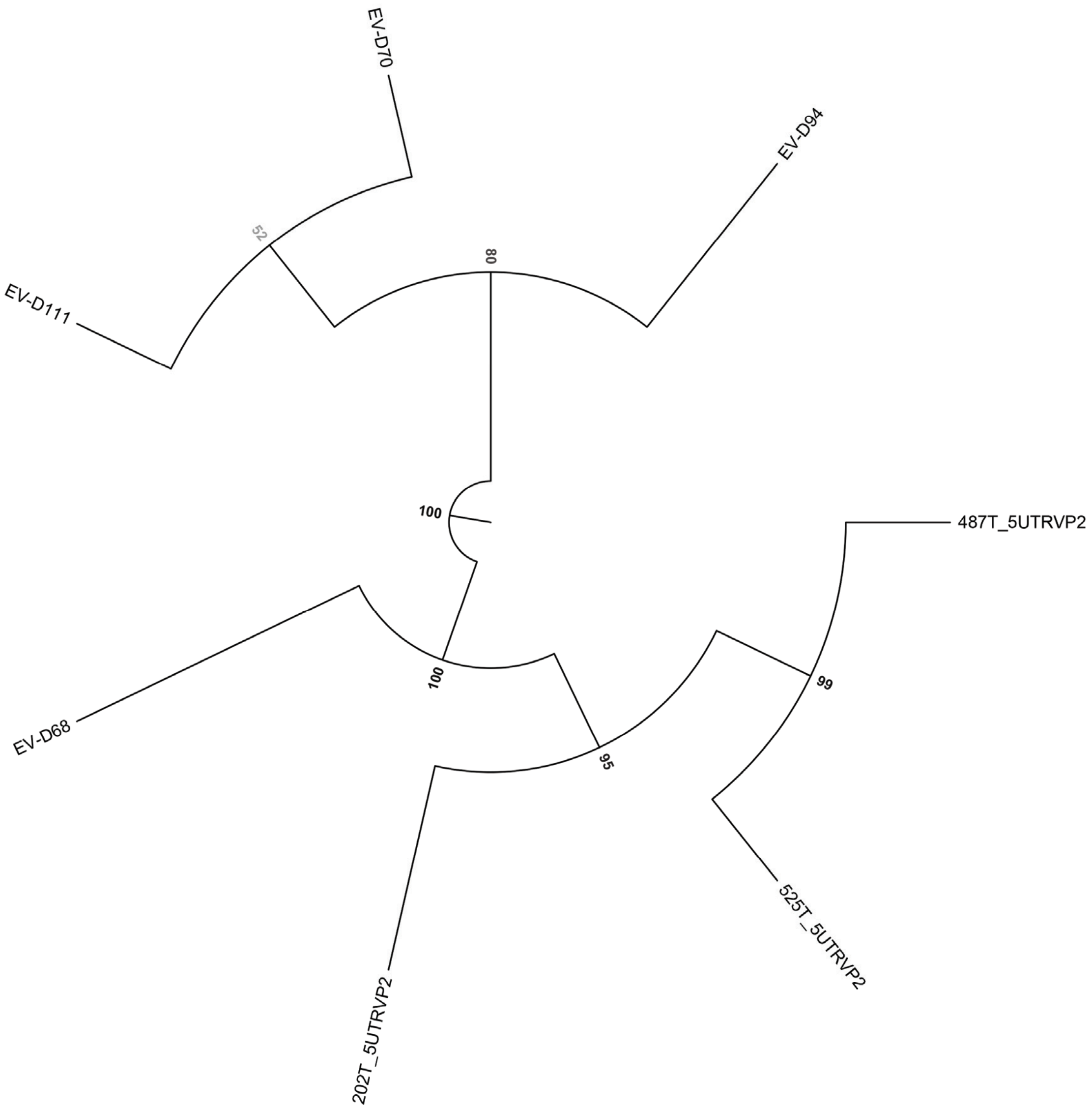

Figure S7. HEV-D 5'UTR/VP2 tree (nt 661 to 993 relative to HRV-A2_X02316).

Reference

1. Tapparel, C.; Junier, T.; Gerlach, D.; van-Belle, S.; Turin, L.; Cordey, S.; Muhlemann, K.; Regamey, N.; Aubert, J.D.; Soccal, P.M., *et al.* New respiratory enterovirus and recombinant rhinoviruses among circulating picornaviruses. *Emerg. Infect. Dis.* **2009**, *15*, 719–726.
2. Linsuwanon, P.; Payungporn, S.; Samransamruajkit, R.; Posuwan, N.; Makkoch, J.; Theanboonlers, A.; Poovorawan, Y. High prevalence of human rhinovirus C infection in Thai children with acute lower respiratory tract disease. *J. Infect.* **2009**, *59*, 115–121.
3. Nix, W.A.; Oberste, M.S.; Pallansch, M.A. Sensitive, seminested PCR amplification of VP1 sequences for direct identification of all enterovirus serotypes from original clinical specimens. *J. Clin. Microbiol.* **2006**, *44*, 2698–2704.
4. Oberste, M.S.; Nix, W.A.; Maher, K.; Pallansch, M.A. Improved molecular identification of enteroviruses by RT-PCR and amplicon sequencing. *J. Clin. Virol.* **2003**, *26*, 375–377.

© 2015 by the authors; licensee MDPI, Basel, Switzerland. This article is an open access article distributed under the terms and conditions of the Creative Commons by Attribution (CC-BY) license (http://creativecommons.org/licenses/by/4.0/).
